# Design of Glycopeptides Used to Investigate Class II MHC Binding and T-Cell Responses Associated with Autoimmune Arthritis

**DOI:** 10.1371/journal.pone.0017881

**Published:** 2011-03-15

**Authors:** Ida E. Andersson, C. David Andersson, Tsvetelina Batsalova, Balik Dzhambazov, Rikard Holmdahl, Jan Kihlberg, Anna Linusson

**Affiliations:** 1 Department of Chemistry, Umeå University, Umeå, Sweden; 2 Medical Inflammation Research, Department of Medical Biochemistry and Biophysics, Karolinska Institute, Stockholm, Sweden; 3 AstraZeneca R&D Mölndal, Mölndal, Sweden; University Hospital Freiburg, Germany

## Abstract

The glycopeptide fragment CII259–273 from type II collagen (CII) binds to the murine A^q^ and human DR4 class II Major Histocompatibility Complex (MHC II) proteins, which are associated with development of murine collagen-induced arthritis (CIA) and rheumatoid arthritis (RA), respectively. It has been shown that CII259–273 can be used in therapeutic vaccination of CIA. This glycopeptide also elicits responses from T-cells obtained from RA patients, which indicates that it has an important role in RA as well. We now present a methodology for studies of (glyco)peptide-receptor interactions based on a combination of structure-based virtual screening, ligand-based statistical molecular design and biological evaluations. This methodology included the design of a CII259–273 glycopeptide library in which two anchor positions crucial for binding in pockets of A^q^ and DR4 were varied. Synthesis and biological evaluation of the designed glycopeptides provided novel structure-activity relationship (SAR) understanding of binding to A^q^ and DR4. Glycopeptides that retained high affinities for these MHC II proteins and induced strong responses in panels of T-cell hybridomas were also identified. An analysis of all the responses revealed groups of glycopeptides with different response patterns that are of high interest for vaccination studies in CIA. Moreover, the SAR understanding obtained in this study provides a platform for the design of second-generation glycopeptides with tuned MHC affinities and T-cell responses.

## Introduction

The development of therapeutic agents that prevent or even reverse disease progression in rheumatoid arthritis (RA) is a challenge in modern drug discovery. RA is an autoimmune disease that affects 0.5–1% of the population, with clinical features including chronic inflammation of peripheral joints and subsequent destruction of cartilage and bone. The disease has been genetically linked to the class II Major Histocompatibility Complex (MHC II) proteins DR1 and DR4 [1]. These proteins bind peptide antigens forming peptide/MHC II complexes (pMHC II) that are presented to circulating CD4^+^ helper T cells, which may initiate an immune response upon activation. In this paper we describe a new methodology, which was applied to the creation of a glycopeptide library used to probe binding to MHC II proteins and subsequent T-cell activation in *in vitro* model systems of human and murine autoimmune arthritis. Structure-based virtual screening was used to enrich sets of biologically active amino acids at two positions in the glycopeptides. The studied amino acids are crucial for binding to MHC II and therefore also crucial for the induction of T-cell responses. The virtual screening was followed by a ligand-based statistical molecular design (SMD) in the amino acid chemical space to select balanced sets of peptides optimal for structure-activity relationship (SAR) analysis.

**Figure 1:**
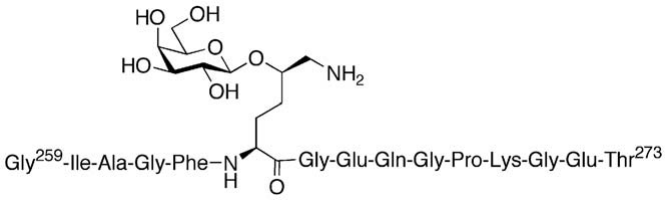
Glycopeptide CII259–273 (1). **1** activates autoimmune T-cells when presented by the MHC II A^q^ protein.

We use collagen-induced arthritis (CIA) [2], a mouse model system, to investigate the role of pMHC II complexes and T-cell activation in RA. Like RA, CIA is genetically linked to expression of a specific class II MHC molecule, *i.e.* the A^q^ protein [3], [4]. It has previously been shown that CII259–273 (**1**, Figure 1), a glycopeptide fragment from type II collagen (CII), can be used as a vaccine to prevent the development of CIA when administered alone [5] or as a complex with solubilized A^q^ protein [6]. Vaccination with the complex also reduces the severity of arthritis in mice with an established chronic relapsing disease. Importantly, glycopeptide **1** has also been linked to RA as it is recognized by T-cell hybridomas generated from transgenic mice expressing human DR4 and the human CD4 co-receptor [7]. Moreover, it elicits responses from T cells isolated from a cohort of RA patients [7]. These findings suggest that it may be possible to reproduce the promising vaccination results observed with CIA in RA patients and thus treat and ultimately cure the disease.

**Figure 2:**
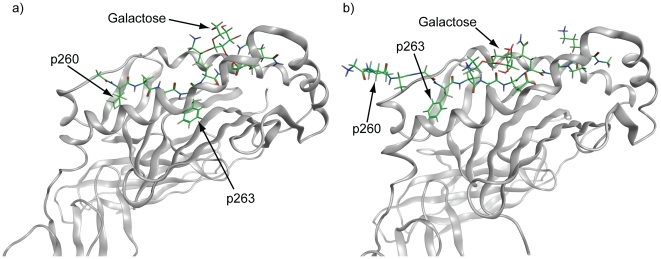
Structural models of the complexes between CII259–270 and A^q^ or DR4. Glycopeptide CII259–270 is bound to the proteins in the characteristic extended conformation with the galactose moiety pointing out from the protein. a) Comparative model of CII259–270/A^q^ [14], [15]. The side chains of Ile^260^ (in p260) and Phe^263^ (in p263) are anchored in the P1 and P4 pockets. P1 is a well-defined, lipophilic pocket of medium size while P4 is a relatively deep, mainly lipophilic pocket. b) Structural model [15] of the complex between CII259–270 and a crystal structure [88] of the DR4 protein. The side chain of Phe^263^ (in p263) is anchored in the DR4 P1 pocket, which is a large, deep and mainly lipophilic pocket. Consequently, Ile^260^ (in p260) is found in the P-3 position on the flanking region of the DR4 binding site.

Glycopeptide **1** binds to the A^q^ protein with the anchor residues Ile^260^ and Phe^263^ positioned in the P1 and P4 pockets of A^q^ (Figure 2a), respectively, while the GalHyl^264^ side chain forms critical interactions with T-cell receptors (TCRs) [8]–[11]. In the present study, the Ile^260^ and Phe^263^ residues have been exchanged for (un)natural amino acids. It has previously been shown that replacing either of these residues with Ala results in strongly reduced affinities for the A^q^ protein [9], [10]. A CII245–270 peptide with the residues Ile^260^, Ala^261^, and Phe^263^ exchanged for Ala, hydroxyproline, and Asp, respectively, has been shown to be a competitive inhibitor of antigen-presentation by A^q^ to T cells [12]. Furthermore, structural modifications of **1** with the introduction of different bioisosteres into the glycopeptide backbone typically resulted in strongly reduced A^q^ binding and T-cell responses [13]–[15]. In conclusion, the information gained prior to this study regarding the ternary TCR/pMHC II complexes involving **1** and A^q^ [8]–[10], [12]–[15] shows that the system is sensitive to structural modifications of the (glyco)peptide, in particular the T-cell response which is often diminished. This observation prompted us to adopt a careful design strategy that combines the strengths of molecular docking and SMD in order to accomplish a set of glycopeptides that could result in a spread in binding affinities to MHC II and with maintained or altered T-cell responses. Such peptides are of interest as candidates for vaccination studies in CIA and potentially in RA. The glycopeptides were primarily designed to target the A^q^ system, but the human DR4 system was also included in the biological evaluations. When **1** binds to DR4 the epitope is shifted so that the side chain of Ile^260^ is found in the P-3 position on the flanking region of the binding site while Phe^263^ occupies the P1 pocket (Figure 2b) [16].

The use of rational structure-based design to develop peptide and peptidomimetic ligands that bind to MHC II has been described earlier in several studies [14], [15], [17]–[23]. The design strategy can be based on intuitive decisions guided by crystal structures or homology models of pMHC II complexes [14], [15], [17]–[21], or it can involve different molecular modeling approaches, *e.g.* pharmacophore matching [22], simulated annealing and/or energy minimizations [18], [24], active-site mapping and *de-novo* peptide design [23]. A few studies have also been presented where molecular docking, *i.e.* a computational method for predicting binding between a ligand and a protein, has been used in redocking experiments [25], [26] and in affinity prediction protocols [27]. In the work presented here, we have applied molecular docking not in retrospect but in the first stage to virtually screen a large set of peptides for their potential to bind to MHC II (A^q^) with preserved epitopes (aiming for retained T-cell responses). The aim was to identify sets of amino acids of interest to include at the anchor positions Ile^260^ and Phe^263^ in glycopeptide **1**.

SMD has been used in ligand-based design of peptides that interact with various protein targets [28]–[30], including MHC proteins [31]. It allows for the identification of amino acid candidates for incorporation into peptides to generate peptide libraries with a balanced range of physicochemical properties, which is important in establishing a reliable SAR. In addition, SMD allows one to minimize the number of peptides that must be synthesized, with maintained statistical robustness in the SAR analysis, when investigating the influence of physicochemical properties on the peptides' bioactivity. This cannot be guaranteed if amino acids have only been changed in one position at a time. We have applied SMD to the resulting amino acid sets from the virtual screening and in the final selection of glycopeptides that, after synthesis and evaluation, gave us novel, informative, and reliable SAR regarding glycopeptide binding to A^q^ and the subsequent T-cell responses. To the best of our knowledge, the study presented in this paper is the first to combine the strengths of molecular docking simulations with SMD to build small but information-rich peptide libraries as tools for robust SAR analysis.

## Methods

### Preparation and characterization of peptide virtual libraries

N^α^-Fmoc protected (*S*)-amino acid derivatives with side chains suitably protected for Fmoc-based solid-phase peptide synthesis were selected from commercial producers. This resulted in 105 amino acid derivatives with non-charged side chains, including both tautomers of His (see File S1). A virtual library of 11025 peptides was constructed from all combinations of the 105 amino acids at the positions 260 (p260) and 263 (p263) in the sequence Ac-Ile^260^-Ala-Gly-Phe^263^-Lys-Gly-Glu-Gln^267^-NH_2_, referred to as the original peptide. The virtual peptide sequences were based on the minimal T-cell epitope with Lys^264^ to reduce the number of rotatable bonds in the dockings since the galactose moiety in the glycopeptide does not significantly affect A^q^ binding [32].

### Tuning of docking software parameters

Software parameters in OMEGA [33] and FRED [34] were tuned to reproduced the original peptide pose observed in the A^q^ comparative model [14]. Design of experiments (DoE) was applied to investigate the influence of adjustable parameters in FRED on the outcome of docking, using full factorial parameter designs [35]. The aim was to find parameter settings that would give robust and acceptable docking results by relating the parameter settings to the root mean square deviation (RMSD) between the original peptide pose and the docking poses obtained of the truncated version of **1** (eight amino acids with 68 heavy atoms and 30 rotatable bonds). The entire tuning process is presented in the File S1.

### Virtual screening, refinement and rescoring

The full library of 11025 peptides was docked to the A^q^ comparative model [14] using FRED [34], with tuned settings and a constraint to restrict the positioning of Lys^264^ (see File S1). The docked peptide conformations were pre-generated using OMEGA [33] with settings tuned according to the findings of Kirchmair et al. [36] (see File S1).

#### Geometrical filtering aiming for maintained or altered T-cell responses

The objective with the study was to design a set of glycopeptides that bind to A^q^ with preserved epitope and thus could elicit a T-cell response. It has been shown that the TCRs are sensitive to structural modifications of **1** [8], [10], [13]–[15]. Thus, we adopted a filtering step where we investigated the geometrical similarity of the docking poses of the 11025 peptides and the original peptide pose. The docked peptides generally exhibited large variations of the backbone conformations at the non-modified C-terminus. Hence, we truncated the peptides after docking to focus the rescoring on the 260–263 fragments (Ac-p260-Ala-Gly-p263-NH_2_). This was motivated by the fact that all peptides share the same C-terminus. The similarity in geometry of the docked pose to the original was assessed in terms of RMSD of the position of the backbone C and N atoms of two poses, calculated using MOE [37]. Poses with RMSD<3.0 Å were energy-minimized in the protein binding cleft using the MMFF94x force field in MOE. After energy minimization, poses with RMSD<1.5 Å were rescored (*i.e.* the binding strengths between the peptides and A^q^ were re-calculated) with the Shapegauss [38], Plp [39], Chemgauss2, Chemgauss3, Chemscore [40], Screenscore [41], and Goldscore [42] scoring functions. Goldscore scoring values were multiplied by −1 for the purpose of comparison with the other scoring functions, with a large negative value corresponding to a high rank.

#### Consensus scoring of individual amino acids aiming for binding to A^q^

The rescoring of the peptides matching the geometrical criteria (see above) was performed to single out peptides with likeliness to display moderate to strong binding to A^q^. A consensus scoring strategy [43], [44] was adopted to reduce the dependency on the individual selected scoring functions [41], [45]–[47]. Consensus scoring can be performed in various ways [44], including multivariate analysis [48], [49], which is the basic strategy we have used here. The novelty in our approach is that we do a statistical-based consensus scoring evaluation for the two varied p260 and p263 separately – *not* on the full peptides. This methodology allow for a selection of sets of amino acids for each of the two varied positions separately that in the next step could be submitted for SMD based on physicochemical properties.

The docking score values for the 260–263 fragments were used to extract information of how well the individual amino acids in p260 and p263 fitted in the A^q^ binding site. The following statistical measures were used for each of the amino acids at the two positions; the frequency (*i.e.* the number of times an amino acid occurred in the culled set of peptides), the best score-value, the average score, and the standard deviation of the score values for each of the used scoring functions. The rescoring thus resulted in two matrices (one for each varied position) where amino acids present in the culled set of peptides were described by 22 parameters - one frequency parameter, and three measures (best score, mean score and standard deviation) times seven scoring functions (Shapegauss [38], Plp [39], Chemgauss2, Chemgauss3, Chemscore [40], Screenscore [41], and Goldscore [42]). The extracted data was visualized with principal component analysis (PCA, see below) to facilitate interpretation and selection. Thus, amino acids selected on the basis of this analysis were highly ranked, had low standard deviations, and appeared frequently in highly scored peptides.

### SMD of glycopeptides

Amino acids selected by the consensus scoring strategy, *i.e.* highly ranked amino acids, were physicochemically characterized by 1D, 2D, and internal coordinate dependent 3D (i3D) molecular descriptors using MOE [37] (see File S1). PCA was used to extract the main principal molecular properties of the two sets of amino acids (p260 and p263, respectively). The resulting principal properties (*i.e.*, the score plots and score vectors) were then used in the selection of amino acids to include in the final set of glycopeptides. Amino acids were selected for p260 and p263 from the respective score plots (the first three principal components) to assure a spread in the chemical diversity that potentially would lead to a spread in biological response and thus enhance the SAR analysis. In this screening stage of the project (*i.e.*, when little was known about the binding preferences of p260 and p263) we decided to include a higher number of amino acids for each position than what was afforded by resource restrictions set by the synthesis if all combinations were to be made. Thus, a final selection of peptides for synthesis was made based on all amino acid combinations (of selected amino acids for p260 and p263) using D-optimal design [50], [51] (see the section on Design of experiments). This small set of glycopeptides contained statistically balanced combinations of the selected amino acids forming a base for a robust SAR analysis. The selected amino acids for p260 and p263 were also described by indicator variables (*i.e.*, qualitative variables) used in subsequent SAR models.

### Principal component analysis

PCA [52], [53] was used to compress and visualize amino acid descriptor data, amino acid docking score values and biological response data by extracting the main variation (*i.e.* principal properties) in the data by calculating the principal components (PCs) and plotting their scores and loadings. The data were mean-centered and scaled to unit variance prior to their use in model calculations. The quality of the model was determined from the proportion of the variation in the original data explained by the model (the cumulative sum of squares of the entries (R^2^X(cum)) and cross-validated cumulative Q^2^ (the cumulative Q^2^(cum) across all PCs)) [54]. Potential outliers were identified using a distance-to-model (DModX) plot. PCA modeling was performed using the Evince software package [54].

### Design of experiments

Factorial designs were employed in the parameter tuning process (see File S1) and D-optimal designs were employed to ensure that the selection of glycopeptides contained physicochemical diversity in the introduced amino acids in p260 and p263. D-optimal design [50], [51] allows for the selection of a subset from a larger collection of molecules such that the subset spans a given property space as thoroughly as possible. Federov's algorithm [55], as implemented in the MODDE software package [56], was used where a D-optimal selection was performed in the PCA property space (the first four score vectors) on combinations of all amino acids selected from PCA score plots (see SMD of glycopeptides) resulting in a final subset of glycopeptides that exhibited physicochemical diversity.

### Relating molecular structure to biological activity using PLS

Partial least square projections to latent structure (PLS) regressions [57], [58] were calculated to interpret the individual amino acids effect on binding to A^q^ or DR4. PLS was used to correlate the indicator variables (*i.e.*, the qualitative description) of the amino acids in p260 and p263 with a response in a **Y** matrix, *i.e.* % inhibition (at 100 μM and 500 μM for A^q^ and DR4, respectively). Here, the peptides were described in terms of the presence or absence of a specific amino acid in p260 and p263. Hence, PLS was used to examine the relationship between 14 variables (seven amino acids in p260 and seven amino acids in p263) and the % inhibition. PLS regression coefficients were analyzed to identify amino acids that resulted in high or low activity. The magnitude of the coefficients was plotted in bar diagrams to visualize the effect of the investigated amino acids. Large positive coefficient indicated that an amino acid was beneficial for high affinity at a specific position while large negative coefficients indicated non-beneficial amino acids. The quality of the model was assessed in terms of the sum of squares of entries for **Y** (R^2^Y) and cross-validation leave-one-out (Q^2^(cum)). The model validity (*i.e.* presence of chance-correlations between the variables and the responses) were determined via 100 permutation experiments [59], [60]. PLS calculations were performed using the SIMCA software package [61].

### Molecular dynamics simulations

MD simulations of A^q^/glycopeptide complexes were performed to investigate if structural or dynamic differences observed in the A^q^/glycopeptide complexes (*i.e.*, the presented epitope) could be linked to the T-cell responses. The initial atomic coordinates were taken from the A^q^ comparative model [14] in complex with the CII259–270 glycopeptide [15]. Only the α_1_ and β_1_ domains of A^q^ (*i.e.* residues α4–α84 and β3–β94) were taken into account in the molecular dynamics (MD) simulations [62]. The side chains of p260 and p263 in the CII259–270 glycopeptide were mutated manually to give the glycopeptides **6**, **7**, and **9**, respectively, and the complexes were energy minimized in two steps using MacroModel within Maestro [63]. First, the complexes were minimized with the protein backbone atoms constrained with a force constant of 100 kJ*mol^−1^*Å^2^ and the maximum number of iterations set to 1000. Second, minimization was performed without any constraints and the maximum number of iterations set to 5000. All other parameters were at their default settings. The Desmond [64], [65] software implemented in Maestro [63] was used for preparing the input structure files for the system while the MD simulation were run using the Desmond MD code implemented on the High Performance Computing Center North (HPC2N) [66]. The minimized complexes were neutralized by adding counter ions (Na^+^) and then solvated using a cubic box shape with a layer of TIP3P water molecules and a salt concentration of 0.15 M NaCl. The distance between the edges of the box and the closest atom in the complex was 15 Å. OPLS-AA force field parameters assigned by Maestro were used in the simulations. The model systems were relaxed prior to the simulations using a default relaxation protocol in Maestro that includes both restrained and unrestrained minimizations followed by four short MD simulations where restraints were gradually removed. A NVT simulation of 18 ns was then performed at 300 K and a recording interval of two ps for both the trajectory and the energy. All other parameters were at their default settings.

### General procedure for solid-phase glycopeptide synthesis

Glycopeptides **2**–**21** were synthesized on Tentagel-S-PHB-Thr(tBu)-Fmoc resin (40 μmol, 0.26 mmol/g) in mechanically agitated reactors. All couplings were performed in DMF and reagent solutions were added to the reactors manually. The indane derivative (2-aminoindane-2-carboxylic acid) is abbreviated to Aic. *N* ^α^-Fmoc amino acids with the following protecting groups were used: *tert*-butyl for Thr, Glu, and Tyr, triphenylmethyl (Trt) for Gln, *tert*-butoxycarbonyl (Boc) for Lys. *N* ^α^-Fmoc amino acids (4 equiv) were activated with 1-hydroxybenzotriazole (HOBt, 6 equiv) or 7-aza-1-hydroxybenzotriazole (HOAt, 6 equiv) and 1,3-diisopropylcarbodiimide (DIC, 3.9 equiv). (5*R*)-*N* ^α^-(Fluoren-9-ylmethoxycarbonyl)-*N* ^ε^-benzyloxycarbonyl-5-*O*-(2,3,4,6-*tetra*-*O*-acetyl-*β*-d-galactopyranosyl)-5-hydroxy-L-lysine [14], [67] (1.5 equiv) was activated with 7-aza-1-hydroxybenzotriazole (HOAt, 3 equiv) and 1,3-diisopropylcarbodiimide (DIC, 1.5 equiv). All coupling reactions were monitored using bromophenol blue [68] as an indicator. The Fmoc protecting group was removed by 20% piperidine in DMF for 10 min. After final Fmoc deprotection, the resin was washed with CH_2_Cl_2_ several times and dried under vacuum. Cleavage of the glycopeptide from the resin and deprotection of acid labile protecting groups was achieved by treatment with trifluoroacetic acid/H_2_O/thioanisole/ethanedithiol (35∶2∶2∶1, 16 mL) for 3 h at 40°C. The resin was filtered off and washed with acetic acid, and the filtrate was concentrated under reduced pressure. The crude peptide was dissolved in acetic acid and concentrated under reduced pressure several times until the concentrated crude peptide was dry. The crude peptide was precipitated, washed with cold anhydrous diethyl ether and lyophilized from a mixture of water and acetic acid (6∶1). It was then purified by preparative reversed-phase HPLC (Beckman System Gold HPLC with a flow rate of 11 mL/min and detection at 214 nm) using a Supelco Discovery® Bio Wide Pore C18 column (250×21.2 mm, 5 μm) and a linear gradient of 0%→100% acetonitrile (0.1% TFA) in H_2_O (0.1% TFA) over 60 min. For analytical reversed-phase HPLC a Supelco Discovery® Bio Wide Pore C18 column (250×4.6 mm, 5 μm) was used instead, with a flow-rate of 1.5 mL/min. After lyophilization, the glycopeptides were deacetylated by treatment with NaOMe in MeOH (20 mM, 1 mL/mg peptide) for 2–3 h at room temperature with monitoring by analytical reversed-phase HPLC. The solution was neutralized by the addition of acetic acid and concentrated under reduced pressure. The residue was purified using reversed-phase HPLC using the conditions described above followed by lyophilization. The final glycopeptide products were ≥95% pure as determined by analytical reversed-phase HPLC. The masses of the peptides were determined by MALDI-TOF mass spectrometry (see File S1).

### MHC-binding assay

The binding of the glycopeptides to A^q^ or DR4 was determined relative to a biotinylated marker peptide in a competitive inhibition assay performed essentially as described elsewhere [10], [69]. Briefly, a mixture of a fixed concentration of purified soluble recombinant MHC II molecules (A^q^ assay: 0.5 μM A^q^; DR4 assay: 1 μM DR4), biotinylated marker peptide at 3 μM (A^q^ assay: CII259–273-bio with Lys^264^; DR4 assay: CLIP-bio with CLIP sequence KPVSKMRMATPLLMQALPM), and various concentrations of competitor glycopeptides **1**–**21** (A^q^ assay: 0, 4, 20, 100, 500 and 2500 μM; DR4 assay: 0, 0.8, 4, 20, 100 and 500 μM) was incubated in PBS containing a protease inhibitor cocktail (Complete™, Boehringer, Mannheim, Germany) at room temperature for 48 h. This mixture (100 μL) was transferred to mAb precoated microtiter assay plates, prepared as described below, and incubated at room temperature for 2 h or at 4°C overnight to capture the MHC II molecules. The plates were washed with PBS containing 0.1% Tween 20 to remove excess peptides and the amount of bound biotinylated marker peptide was detected and quantified by the dissociation-enhanced lanthanide fluoroimmunoassay (DELFIA®) system based on the time-resolved fluoroimmunoassay technique with europium-labeled streptavidin (Wallac, Turku, Finland), according to the manufacturer's instructions. The A^q^ and DR4 experiments were performed in triplicate and duplicate, respectively. Results were reported as % inhibition at a specific concentration since the concentration interval did not support a full dose-response curve, and hence no IC50 values were determined. Microtiter assay plates precoated with mAB were prepared by incubation with 10 μg/mL mAb (A^q^ assay: Y3P 10 mAb; DR4 assay: L243 mAb) at room temperature for 2 h or at 4°C overnight and blocked with PBS containing 2% low fat milk.

### T-cell activation assay

A^q^ [13], [70] and DR4 [7], [8] restricted T-cell hybridomas were used that have been generated previously as reported in the cited literature references. IL-2 production by T-cell hybridomas following incubation with antigen and A^q^- or DR4-expressing antigen-presenting spleen cells was measured in 96-well flat-bottom microtiter plates essentially as described elsewhere [71], but with slight modifications. Briefly, T-cell hybridoma cells (5×10^4^) and A^q^- or DR4-expressing syngeneic spleen cells (5×10^5^) were co-cultured with various concentrations of glycopeptides **1**–**21** (0, 0.01, 0.048, 0.24, 1.2, 6.0, 30, and 150 μM) in a total volume of 200 μL. After 24 h, 100 μL portions of the supernatants were removed, frozen to kill any transferred T cell hybridomas, and assayed for IL-2 production by a sandwich ELISA (capturing mAb, purified rat anti-mouse IL-2; detecting mAb, biotin rat anti-mouse IL-2; PharMingen, Los Angeles, CA) using the DELFIA® system (Wallac, Turku, Finland) according to the manufacturer's instructions. Recombinant mouse IL-2 was used as a positive control.

## Results

A set of 20 glycopeptides was designed using the two-step process consisting of structure-based virtual screening and ligand-based SMD. In the first design stage, docking was applied as a filter to single out peptides from a virtual library with likeliness to bind to A^q^ with a preserved epitope and thus elicit T-cell responses. In the second step, amino acids identified as potential binders were used as a basis for SMD, and a physicochemically diverse subset of amino acid combinations was selected and incorporated at positions p260 and p263 in glycopeptide **1**.

### Structure-based virtual screening of glycopeptides

The virtual library of 11025 anchor-modified analogues of **1**, that included all combinations of 105 amino acids in p260 and p263, respectively, was evaluated in a virtual screening against A^q^ using FRED [34] with the tuned docking software settings. The geometrical filtering of the docked peptides resulted in 1540 of the 11025 peptides that were considered to have a peptide backbone geometrically similar to the original peptide, and thus potentially could present a similar pMHC epitope to the T cells. The 260–263 fragments of these 1540 anchor-modified peptides were subjected to rescoring using seven different scoring functions to estimate the strength of their interaction with A^q^.

**Figure 3:**
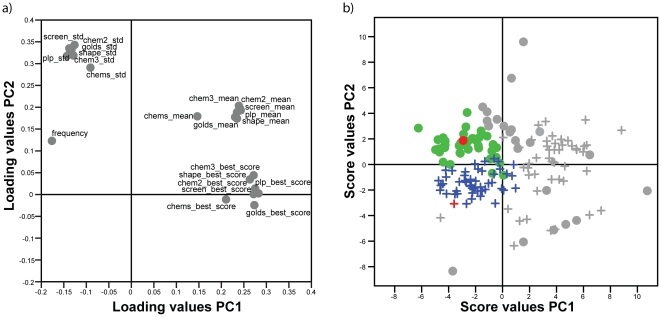
PCA loading and score plots of amino acids score values. a) Loading plot of mean scoring values, standard deviations, top scoring values and frequency of appearance of amino acids present in the docked peptides. b) Score plot of amino acids and their separation due to differences in scoring results. Colored markings indicate selected amino acids. Green dots and blue crosses correspond to amino acids in p260 and p263, respectively, while the red dot and cross correspond to the original amino acids in p260 and p263, respectively.

The adopted consensus scoring approach based on PCA revealed 46 and 52 amino acid substitutions at p260 and p263, respectively, that resulted in highly ranked peptides in the virtual screening (Figure 3 and File S1). The scoring ranks of individual peptides were translated into estimates of the contributions of specific amino acids to the binding (the frequency, the best score, the mean score and the standard deviation for the seven scoring functions - see Methods for details). The PCA had an R^2^X of 0.82 and Q^2^ of 0.75 and loading and score plots for the re-scoring values of the amino acids are shown in Figure 3. Each dot in the score plot corresponds to a unique amino acid present in at least one of the 1540 peptides that remained after the backbone culling steps. The position of each amino acid in the score plot is correlated to its docking score value (a low scoring function value corresponds to a high rank) and frequency of occurrence (*i.e.*, how often that specific amino acid occurred in the culled set of peptides) which are visible in the loading plot in Figure 3a. Hence, amino acids situated to the left in the score plot in Figure 3b were scored highly by the scoring functions and appeared frequently in the 1540 peptides. These amino acids, which are colored green/blue in the score plot (46 and 52 for p260 and p263, respectively, see File S1), were considered to be likely to have affinity for the A^q^ anchoring pockets and were selected for use in the ligand-based glycopeptide design. Notably, the amino acids Ile and Phe at p260 and p263, respectively, present in glycopeptide **1**, were in the highly scored group (Figure 3b). In the case of p260, the amino acids predicted to confer a low affinity for A^q^ (shown on the right in Figure 3b) were either smaller or considerably larger than Ile. For p263, the amino acids associated with low affinity were small and/or aliphatic (*e.g.* Ile) or were considerably more sterically demanding than Phe (*e.g.* biphenyls).

### Ligand-based SMD of glycopeptides

The two selected sets of amino acids for p260 and p263, respectively, with potential to fit the A^q^ binding site *and* to induce a T-cell response, were subjected to the second step in the design process to create a set of glycopeptides with physicochemically diverse amino acids in p260 and p263 optimal for SAR analysis.

**Figure 4:**
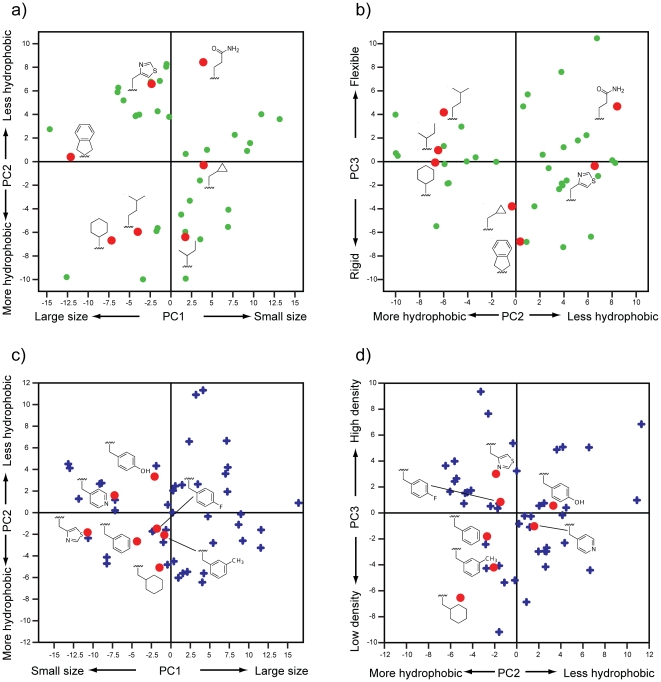
Score plots from PCA models based on amino acid physicochemical properties. Principal properties that dominate each PC are indicated by the axes. Red dots indicate selected amino acids. Green dots and blue crosses indicate unselected amino acids. a) PC1 vs. PC2 for the p260 amino acids. b) PC2 vs. PC3 for the p260 amino acids. c) PC1 vs. PC2 for the p263 amino acids. d) PC2 vs. PC3 for the p263 amino acids.

The physicochemical descriptor data of the 46 and 52 selected amino acids in p260 and p263, respectively, were compressed in two PCA models to identify the main differences and similarities in the amino acids' physicochemical features based on their principal properties. The two models had R^2^X of 0.82 and 0.79, and Q^2^ of 0.73 and 0.67 for p260 and p263, respectively. The principal properties of the p260 and p263 amino acids are shown in the score plots of the three first PCs (Figure 4). For the amino acids selected to fit into the P1 pocket, the principal properties (Figure 4a–b) corresponded to variations in size (*e.g.*, volume, vdW area, molar refractivity), hydrophobicity (*e.g.*, logP, number of hydrogens), and flexibility (KierFlex). The principal properties (Figure 4c–d) of the amino acids selected to fit the P4 pocket corresponded to variation in size (*e.g.* volume, vdW area, rotatable bonds), hydrophobicity (*e.g.*, logP, polar surface areas), and density, respectively. Scores and loading plots for the remaining significant PCs for the p260 and p263 amino acids are presented in the File S1.

**Figure 5:**
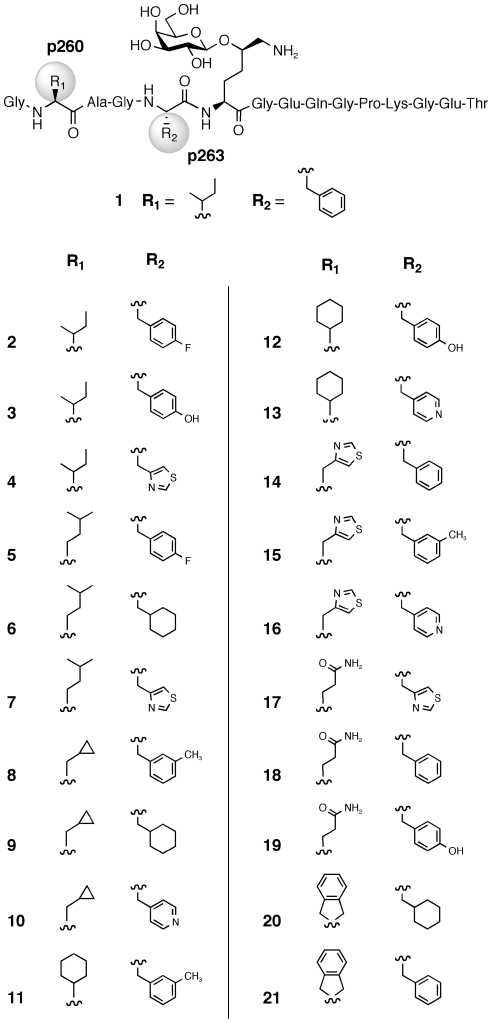
Anchor-modified CII259–273 glycopeptides. Glycopeptides **2**–**21** with modified residues at positions p260 and p263 were synthesized using solid-phase glycopeptide synthesis.

Seven amino acids were selected for p260 and p263, respectively; their side-chains are shown in Figure 5. The selected amino acids exhibit a range of physicochemical properties (*i.e.*, size, hydrophobicity, flexibility and density), while still being in vicinity to the amino acid present in the non-modified glycopeptide. The seven amino acids for p260 and p263, respectively, gave rise to 49 theoretical glycopeptides; statistical design was used to select 20 of these as physicochemically representative peptides for synthesis. Importantly, all of the p260 and p263 amino acids were represented in at least two, and more commonly three, of the 20 peptides, making it possible to draw statistically significant SAR conclusions regarding the effect of individual amino acids and of specific combinations of amino acids in each position.

### Glycopeptide synthesis

The glycopeptides **2**–**21** (Figure 5) with different amino acids in positions p260 and p263 were synthesized using Fmoc-based solid-phase peptide synthesis under standard conditions. After cleavage from the solid support and purification by reversed-phase HPLC the galactose moieties were deacetylated by treatment with methanolic sodium methoxide. Final purification by reversed-phase HPLC then afforded **2**–**21** in 26–51% overall yield based on the capacity of the resin. All of the glycopeptides had a purity of ≥95% according to analytical reversed-phase HPLC and their structures were confirmed by MALDI-TOF mass spectrometry.

### Binding to A^q^

The binding of glycopeptides **1**–**21** to A^q^ was evaluated using a competitive ELISA-based assay in which the glycopeptides, in a range of concentrations, were incubated with recombinant soluble A^q^ protein and a fixed concentration of a biotinylated marker peptide. Of the 20 designed glycopeptides, 11 were classified as actives in a preliminary run and were included in a second run where dose-dependent inhibition curves were obtained (Table 1 and File S1). The inhibitory effects of the anchor-substituted glycopeptides ranged from 20–87% at 100 μM, compared to 90% inhibition for the non-modified **1**. Two glycopeptides (*i.e.* **2** and **4**) were found to have comparable affinity for A^q^ to that of the non-modified **1** while the other nine glycopeptides that also bound to A^q^ displayed 20–55% inhibition. Five out of the seven selected amino acids for p260 and p263, respectively, displayed binding to A^q^ and the SAR analysis of the A^q^ glycopeptide binding data revealed structural elements within the modified amino acids that were important for binding.

**Table 1:** Competitive inhibition of biotinylated CII259–273 binding to the A^q^ protein by glycopeptides **1**–**21** and responses by A^q^-restricted T-cell hybridomas.

	A^q^ binding^a^		T-cell responses^b^					
Pept.	% inhibition (100 µM)	% inhibition (500 µM)	22a1-7E	HCQ.3	HCQ.10	HM1R.2	HD13.9	HNC.1
**1**	90±1	97±0	++++	+++++	++++	++++++	+++++	+++++
**2**	87±1	95±0	++++	+++	+++	+++++	++++	++++
**3**	28±3	59±3	+	+++	+	+++	++	++
**4**	86±1	94±0	+++	+++++	++	+++++	++++	++
**5**	29±2	58±2	++	+	+	++	+++	++
**6**	46±1	75±0	−	−	+	++	−	−
**7**	39±2	67±1	+	+++	−	+++	++	++
**8**	55±3	78±1	+	+	+	+++	−	−
**9**	38±6	70±1	−	−	−	+	−	−
**10**	−	−	n.d.	n.d.	n.d.	n.d.	n.d.	n.d.
**11**	29±3	53±8	+	+	+	++	+	−
**12**	−	−	n.d.	n.d.	n.d.	n.d.	n.d.	n.d.
**13**	−	−	n.d.	n.d.	n.d.	n.d.	n.d.	n.d.
**14**	20±4	50±8	+	+++	+	+++	++	−
**15**	41±1	71±2	−	−	++	++	−	−
**16**	−	−	n.d.	n.d.	n.d.	n.d.	n.d.	n.d.
**17**	−	−	n.d.	n.d.	n.d.	n.d.	n.d.	n.d.
**18**	−	−	n.d.	n.d.	n.d.	n.d.	n.d.	n.d.
**19**	−	−	n.d.	n.d.	n.d.	n.d.	n.d.	n.d.
**20**	−	−	n.d.	n.d.	n.d.	n.d.	n.d.	n.d.
**21**	−	−	n.d.	n.d.	n.d.	n.d.	n.d.	n.d.

a Data are expressed as the percentage of biotinylated CII259–273 bound in the absence of competitor glycopeptide (mean values of triplicates ± one standard deviation). Glycopeptides assigned with – were classified as inactive (<30% inhibition) in a previous assay and were not included in this assay (see File S1).

b The magnitude of the T-cell responses were determined from the concentration of antigen required to induce secretion of IL-2 corresponding to 10% of the measured max response for glycopeptide **1**: − = no response, + = 150 µM, ++ = 30 µM, +++ = 6.0 µM, ++++ = 1.2 µM, +++++ = 0.24 µM, ++++++ = 0.0064 µM, n.d. = not determined. T-cell hybridomas were selected from groups with different specificity for the hydroxyl groups on the GalHyl^264^ moiety [8].

**Figure 6:**
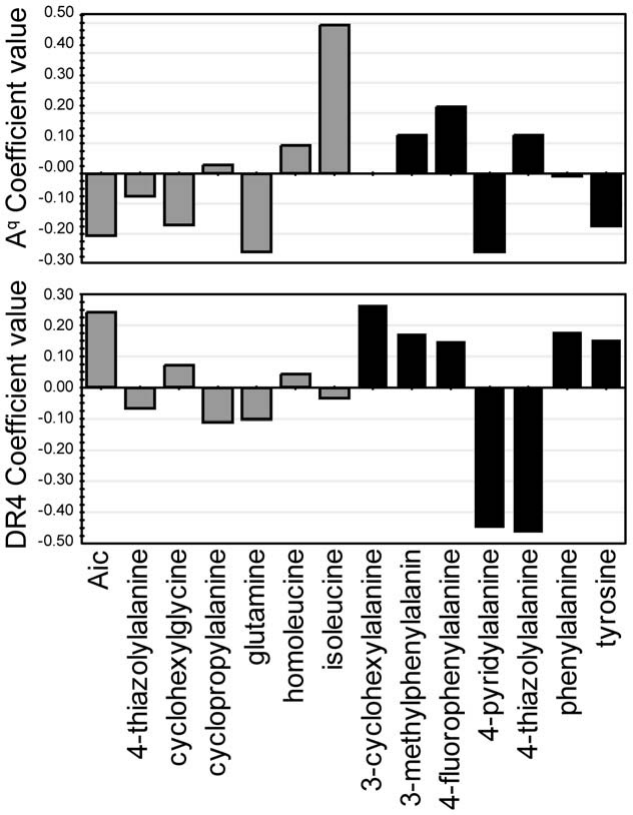
PLS regression coefficient plots. The PLS models were created by relating glycopeptide properties (expressed via the presence of a specific amino acid) and their binding capacity to A^q^ (upper plot) and DR4 (lower plot) at peptide concentrations of 100 µM and 500 µM, respectively. A large positive coefficient indicates that the amino acid was beneficial for a high affinity. Grey bars represent p260 while black bars represent p263. The indane derivative (2-aminoindane-2-carboxylic acid) is abbreviated to Aic.

The SAR analysis was facilitated by the linear PLS-regression model correlating the presence of a specific amino acid in p260 and p263 to the affinity of the peptides for A^q^ (with model statistics R^2^Y of 0.83, Q^2^ of 0.52). Permutation experiments indicated a low risk of chance-correlations (see File S1). The regression coefficients (Figure 6) provided a direct interpretation of the effect that the different amino acids had on the binding to the MHC II proteins.

#### P1 binding preferences

P1 in the A^q^ binding site is a medium-sized and relatively lipophilic pocket that accommodates Ile^260^ of the non-modified **1**, as shown in Figure 2. Analysis of the coefficient values from the PLS regression model based on the A^q^ binding data clearly showed that Ile was the preferred amino acid in p260 (upper plot in Figure 6). Accordingly, glycopeptides **2** and **4**, which both contained Ile in p260, were the only anchor-modified glycopeptides that had comparable A^q^ affinity to that of **1** (Table 1). As reflected in the A^q^ coefficients in Figure 6, introduction of either larger side chains, *e.g.* homoleucine (*cf.* **5** with **2**, **7** with **4** in Table 1) and cyclohexylglycine (*cf.* **12** with **3**), or a smaller side chain, *i.e.* cyclopropylalanine (*cf.* **6** with **9**), led to reduced A^q^ binding. Substitution of Ile with 4-thiazolylalanine also led to significant loss of A^q^ affinity (*cf.* **14** with **1**). Gln and the indane derivative that had the most negative A^q^ coefficients (Figure 6) resulted in complete loss of binding. Thus, of the amino acids examined in p260, Ile was found to be the best choice suggesting that the glycopeptide side chain inserted in the P1 pocket should preferably be of moderate size and bulkiness, aliphatic, and flexible.

#### P4 binding preferences

The P4 pocket in the A^q^ binding site has a larger volume and is deeper than the P1 pocket and it accommodates the side chain of Phe^263^ of the non-modified **1**, as shown in Figure 2. Interestingly, the coefficient values in the PLS regression model based on the A^q^ binding data suggest that three of the introduced substitutions in p263 were more beneficial for A^q^ binding than the original Phe (upper graph of Figure 6). These were the 4-fluorophenylalanine (*cf.* **2** with **1** in Table 1), 4-thiazolylalanine (*cf.* **4** with **1**), and *m*-methylphenylalanine (*cf.* **15** with **14**), which all had larger positive coefficients than Phe. 3-Cyclohexylalanine seems to be tolerated equally well as Phe (*cf.* **6** with **5** and **7**). In contrast, strongly negative effects were observed for tyrosine and 4-pyridylalanine. Replacing Phe with tyrosine led to a loss in binding to A^q^ (*cf.* **3** and **19** with **1**) and 4-pyridylalanine was not tolerated at all as it led to a complete loss of A^q^ binding for the investigated glycopeptides (see **10**, **13** and **16**).

### Recognition by A^q^-restricted T-cell hybridomas

The ability of the designed glycopeptides to induce T-cell responses was studied using a panel of A^q^-restricted T-cell hybridomas [13], [70] specific for **1**. Antigen-presenting spleen cells in the presence of various concentrations of glycopeptides **1**–**21** were incubated with T-cell hybridomas selected from groups previously established to have different specificities for the hydroxyl groups on the GalHyl^264^ moiety [8]. Interleukin-2 (IL-2) secreted into the medium upon recognition of the MHC II/glycopeptide complex by a T-cell hybridoma was then quantified in an ELISA. Only glycopeptides that bound to A^q^ were evaluated for T-cell recognition.

The 12 glycopeptides evaluated were found to induce different T-cell response profiles (Table 1 and File S1). Glycopeptides **2** and **4** that bound as strongly to A^q^ as the non-modified **1** (Table 1), did also induce medium to strong responses from most of the T-cell hybridomas. Among the glycopeptides that bound with medium affinity to A^q^ (20–55% inhibition), two subgroups could be distinguished with respect to their T-cell responses. One subgroup induced weak to medium T-cell responses from most of the hybridomas (*cf.* **3**, **5**, **7** and **14**) while another subgroup induced weak responses usually from only a few of the hybridomas (*cf.* **6**, **8**, **9**, **11**, and **15**). The differences in T-cell responses between these two subgroups could not be linked to different affinity to A^q^. For example, **7** and **9** had similar binding to A^q^, but while **7** stimulated all hybridomas except HCQ.10 moderately, **9** was weakly recognized only by the HM1R.2 hybridoma. A common feature of the glycopeptides that bound with medium affinity to A^q^, but induced weak or non-existent T-cell responses, was the presence of a large side chain in p263, *i.e.* cyclohexylalanine in **6** and **9** and *m*-methylphenylalanine in **8**, **11**, and **15**.

### Molecular dynamics simulations of A^q^/glycopeptide complexes

The MD simulations of the A^q^/glycopeptide complexes performed to investigate if structural or dynamic differences of the presented epitope could be linked to the T-cell responses were focused on selected glycopeptides (**1**, **6**, **7** and **9**). Glycopeptides **6**, **7** and **9** had non-natural amino acids in p260 and p263 (Figure 5) and comparable affinity for A^q^ (38–46% at 100 μM), but displayed different T-cell response patterns. Glycopeptide **7** generally induced medium T-cell responses, *i.e.* responses that correlated well with a somewhat weaker A^q^ binding, while both **6** and **9** elicited weak or no T-cell responses despite having medium affinity for A^q^ (see Table 1). The MD simulations revealed that the variation in RMSD values for the whole A^q^/glycopeptide complex was larger for the glycopeptides that induced medium/strong T-cell responses (**1** and **7**) than for glycopeptides that induced weak/no T-cell responses (**6** and **9**), suggesting that some degree of flexibility was important for the ability to elicit a T-cell response (File S1). Interestingly, **6** and **9** also revealed structural differences between each other. Glycopeptide **6** was characterized by having a 1.2–1.7 Å shorter distance between the Cα of GalHyl^264^ and the A^q^ protein as compared to the other complexes (File S1). This indicated that the epitope of **6** presented to the TCRs could be slightly altered, *e.g.* by binding of the glycopeptide deeper in the A^q^ binding site. Glycopeptide **9** instead had a smaller variation in RMSD values for the ligand main chain atoms than glycopeptides **1**, **6**, and **7**, suggesting that it is more firmly anchored in the A^q^ binding site (see File S1).

### Binding to DR4 and recognition by DR4-restricted T-cell hybridomas

Binding to the human DR4 protein was also evaluated using a competitive ELISA-based assay. All of the designed glycopeptides (**2**–**21**) generated dose-dependent inhibition curves and their inhibitory effects ranged from 19 to 84% at 500 μM, compared to 72% inhibition for the non-modified glycopeptide **1** (Table 2). The P1 pocket in the DR4 binding site that accommodates the side chain of Phe^263^ in **1** is a large, deep and mainly lipophilic pocket (Figure 2b).

**Table 2:** Competitive inhibition of biotinylated CLIP binding to the DR4 protein by glycopeptides **1**–**21** and responses by DR4-restricted T-cell hybridomas.

	DR4 binding		T-cell responses	
Pept.	% inhibition(100 µM)	% inhibition(500 µM)	mDR17.2	hDR11.2
**1**	39±12	72±4	+++	+++
**2**	45±5	73±6	++++	++++
**3**	41±2	65±4	++++	+++
**4**	8±4	31±10	+++	−
**5**	49±4	77±1	+++++	++++
**6**	62±2	84±4	+++++	+++
**7**	10±14	34±6	+++	−
**8**	50±1	70±3	+++++	++++
**9**	44±2	75±0	+++++	+++
**10**	20±27	19±15	++	−
**11**	51±5	76±5	+++++	++++
**12**	48±2	73±3	++++	+++
**13**	22±14	52±3	+++	+
**14**	46±11	74±1	++++	+++
**15**	55±2	76±2	+++++	++++
**16**	18±22	23±11	+	−
**17**	20±10	26±11	++	−
**18**	47±9	60±6	+++	+++
**19**	37±8	80±5	+++	+++
**20**	62±2	82±3	++++	++
**21**	44±1	84±5	++++	++++

a Data are expressed as the percentage of biotinylated CLIP bound in the absence of competitor glycopeptide (mean values of triplicates ± one standard deviation).

b The magnitude of the T-cell responses was determined from the concentration of antigen required to induce secretion of IL-2 corresponding to 10% of the measured max response for the native CII259–273 glycopeptide **1**: − = no response, + = 150 µM, ++ = 30 µM, +++ = 6.0 µM, ++++ = 1.2 µM, +++++ = 0.24 µM, ++++++ = 0.0064 µM. T-cell hybridomas were selected from groups with different specificity for the hydroxyl groups on the GalHyl^264^ moiety.

As in case for A^q^, a linear PLS-regression model correlating the presence of a specific amino acid in p260 and p263 to the affinity of the peptides for DR4 was also established (with model statistics R^2^Y of 0.90, Q^2^ of 0.55). Permutation experiments again indicated a low risk of chance-correlations (see File S1).

The regression coefficients of the PLS-model based on DR4 binding data showed that four of the seven amino acids introduced in p263 were well tolerated (lower part of Figure 6). 3-cyclohexylalanine, *m*-methylphenylalanine, 4-fluorophenylalanine, and Tyr were equally or more beneficial for DR4 binding as compared to Phe. In contrast, glycopeptides with 4-pyridylalanine or 4-thiazolylalanine in p263 bound poorly to DR4 (Figure 6 and Table 2). Amino acids introduced in p260, located in the P-3 position on the flanking region of the binding site (Figure 2), did not have a large influence on DR4 binding. However, a trend was noted where larger and more hydrophobic amino acids seemed beneficial since, for example, the indane derivative improved binding somewhat (*i.e.*, **20** and **21** in Table 2).

DR4-restricted T-cell responses were evaluated with hybridomas mDR17.2 and hDR11.2 [7], [8] specific for **1** using a similar assay setup as for the A^q^ system (Table 2). Generally, all glycopeptides that bound well to DR4 (60–84% inhibition at 500 μM) also produced strong T-cell responses with both hybridomas. Interestingly, glycopeptides **5**, **8**, **11**, and **15** that bound strongly to DR4 with affinities comparable to **1** generally elicited even stronger T-cell responses than **1**. Glycopeptides with thiazolylalanine and 4-pyridylalanine in p263 that bound weakly to DR4 (19–52% inhibition at 500 μM) generally elicited weaker T-cell responses and, in particular, the hDR11.2 hybridoma typically failed to respond.

### Response patterns displayed by the modified glycopeptides

We investigated the similarities and differences between the glycopeptides with respect to MHC II binding and recognition by different T-cell hybridomas. An investigation of the responses collectively is attractive from a drug (vaccine) development perspective as the identification of peptides with similar response patterns in A^q^ and DR4 systems provides chemical probes that can be used to link the pharmacodynamic, pharmacokinetic and toxicity profiles monitored in the A^q^ mouse model of CIA to DR4-based models. The latter is more closely associated with development of RA in humans, in particular from a MHC II perspective.

**Figure 7:**
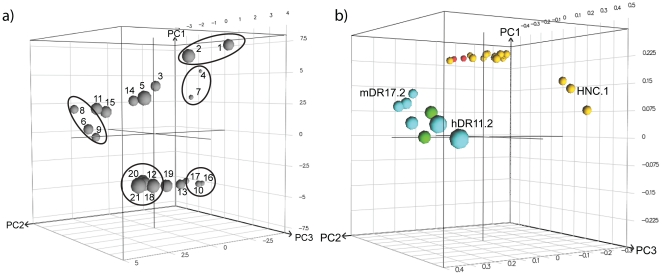
Response pattern displayed by the glycopeptides. The response pattern is visualized by score and loading plot from PCA based on the results from the A^q^ and DR4 binding and T-cell recognition assays. a) 3D score plot of PC1, PC2 and PC3 displaying similarities and differences between glycopeptides depending on response patterns. Circles indicate groups among the glycopeptides discussed in the text. b) Loading plot of the biological responses. A^q^ and DR4 binding at 100 µM and 500 µM are indicated by red and green spheres, respectively. A^q^-restricted hybridomas 22a1-7E, HCQ.3, HCQ.10, HD13.9, HM1R.2 and HNC.1 and DR4-restricted hybridomas mDR17.2 and hDR11.2 at concentrations 150, 30 and 6 µM are indicated by yellow and blue spheres, respectively. Hybridomas specifically discussed in the text are indicated by their names.

A PCA was constructed based on all biological responses and a score plot is presented in Figure 7a displaying the response patterns of the glycopeptides, where those that are close to each other have similar biological activity fingerprints (*i.e.*, similar affinities for A^q^ and DR4, and similar T-cell response patterns). The loading plot in Figure 7b displays the contributions from the different responses that give rise to the patterns seen in the score plot. The glycopeptides are differentiated in PC1 mainly by their different affinity for A^q^ and their recognition by A^q^-restricted T-cell hybridomas, thereby forming two groups of peptides; binders (high score values) and non-binders (low score values). From the loading plot, it is clear that the responses from all A^q^-restricted hybridomas, except HNC.1, are well correlated with the glycopeptides A^q^ binding strength. The difference in ability of the glycopeptides to bind to DR4 and be recognized by DR4-restricted hybridomas is illustrated in PC2, where active glycopeptides have a high score-value. Deviations in the glycopeptides regarding their strength of A^q^ and DR4 binding and the magnitude of recognition by the A^q^-restricted hybridoma (*e.g.* HNC.1) and the DR4-restricted hybridomas (*e.g.* hDR11.2 and mDR17.2), respectively, were the main information imbedded in PC3.

Glycopeptide **2** stands out as having similar activity fingerprints as **1**, since it binds well to A^q^ and DR4 and induces strong T-cell responses. Glycopeptides with affinity for both proteins included **6**, **8** and **9**, but these were poorly recognized by the A^q^-restricted hybridomas. Glycopeptides **4** and **7** are A^q^-specific, *i.e.* they both displayed affinity for A^q^ but bound poorly to DR4. Among the glycopeptides with poor binding to A^q^, seen in the lower regions of the score plot in Figure 7a, glycopeptides **18**, **21**, **20** and **12** were DR4-specific while **13**, **17**, **10** and **16** did not bind well to either protein, nor gave a good T-cell response.

## Discussion

In this work a careful design strategy was adopted that combined the strengths of molecular docking and SMD in order to accomplish a set of glycopeptides with varying binding affinities for A^q^ giving rise to maintained or altered T-cell responses. The synthesis and biological evaluation of these designed glycopeptides should give rise to glycopeptides that could be useful in the future development of a vaccine. Since the design efforts were extensive one may ask: was this design strategy successful and worth the efforts? One way to address this question would be to link the initial aim of the study with the final biological results, in the context of the design strategy.

The biological evaluation revealed that 11 out of the 20 modified glycopeptides (five different amino acids in p260 and p263, respectively) bound to A^q^ with a satisfying spread in the inhibitory effects ranging from 20 to 87% at 100 μM. These results should be contemplated while keeping in mind that no extensive SAR has previously been published for the two investigated anchor positions (p260 and p263). The presented SAR analysis of glycopeptides binding to A^q^ showed that Ile was the preferred amino acid in p260 since the P1 pocket was sensitive to all other substitutions. In contrast, the P4 pocket accepted most of the introduced modifications such as Phe derivatives with an electron-withdrawing *para*-fluoro or electron-donating *meta*-methyl substituent, 3-cyclohexylalanine, or a thiazole derivative. The A^q^ comparative model indicated that the lower half of the P1 and P4 pockets contain a hydrophilic region due to the presence of a Glu and a Ser residue, respectively. As mentioned earlier, one previous study has presented that Asp in p263 could bind to A^q^ [12]. In the present study, modified glycopeptides with Gln or thiazolylalanine in p260 had poor affinity for A^q^ indicating that no favorable interactions in the hydrophilic regions in P1 were formed. In the p263 position, on the other hand, glycopeptides with *para*-fluorophenyl- and thiazolylalanine showed good affinity for A^q^, while pyridylalanine and tyrosine had no or weak affinity indicating that the P4 pocket is a potential site for optimization of electrostatic interactions with the glycopeptides.

As many as 10 modified glycopeptides gave T-cell responses for more than one A^q^ restricted hybridoma. We consider this a step forward in the project as previous information gained regarding the ternary TCR/pMHC II complexes [8]–[10], [12]–[15] has revealed that the system is sensitive to structural modifications of the (glyco)peptide, with diminished T-cells responses as a consequence. The results regarding T-cell responses and its implication for vaccination studies are considered in more detail in the final part of the Discussion.

An interesting outcome of the biological results was the information regarding DR4 binding and subsequent T-cell responses. Although docking into DR4 was not included in the design process, the set of 20 glycopeptides had an inhibitory effect of 19 to 84% at 500 μM on DR4, and all elicited a T-cell response. Furthermore, a SAR for p260 and p263 of glycopeptide **1** could be established for binding to DR4, just as for A^q^. Although the P4 pocket of A^q^ and the P1 pocket of DR4 are similar due to high sequence identity in the binding sites, different amino acids were beneficial in the binding to the different proteins. We hypothesize that the informative results obtained for DR4 could be a direct effect of the design strategy. Instead of making a stringent focused library towards optimizing binding to A^q^, the design strategy presented here adopted a more “loose” approach where amino acids that are less likely to bind A^q^ and induce a T-cell response were culled. The “surviving” amino acids were subjected to an SMD based on physicochemical properties. This appears to have generated a set of glycopeptides that provided information not only for A^q^ (that was used in the design) but also to other related target proteins, as DR4.

Most of the modifications that we introduced in p263 proved to be well-tolerated in binding to DR4 and recognition by DR4-restricted T-cell hybridomas. These findings are consistent with previous studies where the DR4 P1 pocket was challenged with several peptides substituted at the MHC anchor position using different (un)natural amino acids [22], [72], [73]. In the cited studies even larger side chains than those explored in our study were successfully introduced. However, we noted that Tyr was a good substitution whereas it has previously been reported to decrease DR4 affinity 3-fold for CII261–273 [16]. Also we found that the thiazole and pyridine derivatives exhibited a reduced affinity for DR4 and a weak or non-existent T-cell response. This is interesting since the Phe^265^ of the human cartilage glycoprotein-39(263–275), which also is assumed to bind in the DR4 P1 pocket, has successfully been replaced with both 2-thienylalanine and 3-pyridylalanine [72].

If we return to the initial question: was the extensive design strategy based on molecular docking and SMD successful and worth the efforts? Such question is always difficult to answer, but if the design efforts are put into perspective of the information gained for the A^q^ and DR4 systems, we judge the design successful. The presented work also resulted in glycopeptides that now are submitted to vaccination studies (see detailed discussion below). The current study was conducted on MHC II systems, but it would be interesting to see the design strategy extended to more systems to investigate its general applicability.

Variations in T-cell responses elicited by peptide ligands with modified MHC anchor residues have been described previously [74]–[80]. For example, peptides with modified MHC anchor residues that show decreased affinity for I-E^k^ have been identified to function as partial agonists of the TCR [76]. MHC anchor-modified peptides have also been shown to induce anergy in polyclonal T-cell populations [77], [78], and in one of the studies it was reported that when administered *in vivo* to mice, the peptide reduced both the severity and incidence of EAE as well as being able to ameliorate an already established disease [77]. In this study, the strengths of the A^q^-restricted T-cell responses elicited by the modified glycopeptides generally correlated well with the glycopeptides affinities for A^q^. However, some glycopeptides had similar A^q^ binding strengths but induced significantly different T-cell responses. We observed that larger side chains in p263, *i.e.* cyclohexylalanine and *m*-methylphenylalanine, were generally associated with weak or non-existent T-cell responses despite medium affinity to A^q^.

In the present study MD simulations of selected A^q^/glycopeptide complexes that displayed similar affinity for A^q^ but differed in their T-cell responses revealed structural differences between the two subgroups. Thus, a lower degree of flexibility in the complex, or binding of the glycopeptides deeper in the A^q^ binding site, appeared to be correlated with weak or non-existent T-cell responses. Small structural differences has previously been observed in X-ray crystal structures of E^k^/peptide complexes where substitution of an MHC anchor residue with maintained MHC binding resulted in a 1000-fold decreased T-cell response [74]. Various authors have suggested that differences in the rate of dissociation of the complex formed between the pMHC and the TCR might be attributable to structural changes in the pMHC complex that result in altered TCR recognition [81]–[84]. Generally, faster dissociation rates have been found for interactions that lead to a partially agonistic response while slower dissociation rates are associated with interactions that lead to a fully agonistic response. Differences in the rate of dissociation could also account for the differences in TCR signaling observed for the modified glycopeptides presented in this study.

Depending on the strategy chosen for a vaccination study using CIA as a model for RA, glycopeptides with different MHC binding and T-cell response profiles (cf. Figure 7) will be of interest. If complexes between the peptide and class II MHC are used for *in vivo* vaccination it is most likely beneficial to use a glycopeptide with high MHC affinity that is also recognized well by the disease-promoting T cells (*e.g.* glycopeptide **2** is an interesting candidate for vaccination in mice expressing A^q^, just like **1**). Since the complex does not give a co-stimulatory (second) signal to the T cells anergy should be induced. Alternatively, such peptides could have an altered T-cell recognition, which may address other T-cell clones with regulatory functions. If instead the glycopeptide alone is used for treatment of an already established disease, it will bind to class II MHC on professional antigen-presenting cells that potentially are capable of activating disease-promoting T cells since they also provide a second, co-stimulatory signal. It could then be an advantage to use a peptide that binds weaker to the MHC (*e.g.* glycopeptide **7** in A^q^ expressing mice), or one that binds well but induces a weak or altered T-cell response, so as not to make an ongoing disease worse. For example, it has previously been reported that CII-based peptide analogs with modified MHC anchors that bound poorly to the MHC proteins can suppress CIA in A^q^-expressing mice as well as in DR1- and DR4-transgenic mice [12], [75], [85]–[87]. Especially if the glycopeptide alone is used for vaccination it would be beneficial to improve its metabolic stability by *e.g.* non-natural amino acids to increase the glycopeptide's half-life and thereby its therapeutic effect.

Another important issue that one has to consider in the development of a vaccine for RA is that the results conducted on mice needs to translate to humans. This challenging task will have a greater potential of success if one candidate drug shows the same response pattern in several species, including humans. Hence, it is important to explore compounds that show both species-specific and cross-species activity early in the process. For example, glycopeptides such as **2**, which bind well to both A^q^ (present in mice) and DR4 (present in humans) and induces strong T-cell responses, could just like **1** provide an understanding of how effects obtained in the A^q^ mouse model translate to the DR4-based transgenic RA model. We also identified groups of glycopeptides that bound only to A^q^ (*e.g.* **4** and **7**), another group that bound only to DR4 (*e.g.* **12** and **21**). All theses glycopeptides may be valuable in species-specific studies to elucidate immunological effects that could further be used in the development of a vaccine for RA.

### Conclusions

In this work, a powerful combination of molecular docking and SMD was used to study glycopeptide receptor interactions so as to obtain SAR understanding from a minimized set of glycopeptides. Selection of the minimized set was done by filtration using structure-based design (molecular docking) were glycopeptides likely to fit the receptor binding site with preserved epitope were identified. This filtration was followed by SMD, where peptides with diverse physicochemical properties were selected, which in turn led to a variation in their receptor affinities. The strategy proved successful when applied to the glycopeptide CII259–273 from type II collagen and led to novel glycopeptides that bound with variable affinity to the class II MHC A^q^ and DR4 receptor proteins. Rational SAR conclusions could be drawn which revealed the binding preferences of the anchoring pockets in the two MHC proteins and SAR models were established relating the glycopeptides' properties to their affinities for A^q^ and DR4. Several glycopeptides induced strong responses in panels of T-cell hybridomas, selective for complexes of CII259–273 with A^q^ or DR4, while others induced weak to medium T-cell responses, thereby providing a high-level SAR understanding. An analysis of all of the responses, including binding to A^q^ and DR4 as well as recognition by T-cell hybridomas, revealed groups of glycopeptides with different response patterns, from which candidates for *in vivo* vaccination studies in CIA can be selected. Moreover, the SAR understanding obtained in this study provides a platform for design of second-generation glycopeptides, *e.g.* glycopeptides that bind to MHC with higher affinity, or that generate fine-tuned T-cell responses. Finally, it should be highlighted that the design strategy presented in this work is applicable in the design of new ligands for any protein-ligand system where a structural model of the complex is available.

## Supporting Information

File S1 Contents. Amino acid descriptors. Amino acid derivatives incorporated into virtual peptides at positions p260 and p263. Docking software parameter tuning and constraints. Table of amino acids selected for p260 and p263 based on docking scores. Score and loading plots from the PCA based on physicochemical properties of amino acids in p260 and p263. Table with yields. purity and MALDI-TOF data for the synthesized glycopeptides. HPLC chromatograms for the modified glycopeptides. Initial A^q^ binding affinity assay. Dose-response curves for binding to A^q^. Dose-response curves for A^q^-restricted T-cell responses. Dose-response curves for binding to DR4. Dose-response curves for DR4-restricted T-cell responses. PLS Permutations experiments. RMSD vs. simulation time plots for the whole complex and the ligand. The distance between the GalHyl^264^ CA and the A^q^ Lys11C as a function of the simulation time. References.
(PDF)
