# Supplementary material for: Design of Glycopeptides Used to Investigate Class II MHC Binding and T-Cell Responses Associated with Autoimmune Arthritis
Source: PLoS One. 2011 Mar 15;6(3):e17881. doi: 10.1371/journal.pone.0017881 (PMC3058040; doi:10.1371/journal.pone.0017881)
Supplement: File S1 — Contents. Amino acid descriptors. Amino acid derivatives incorporated into virtual peptides at positions p260 and p263. Docking software parameter tuning and constraints. Table of amino acids selected for p260 and p263 based on docking scores. Score and loading plots from the PCA based on physicochemical properties of amino acids in p260 and p263. Table with yields. purity and MALDI-TOF data for the synthesized glycopeptides. HPLC chromatograms for the modified glycopeptides. Initial Aq binding affinity assay. Dose-response curves for binding to Aq. Dose-response curves for Aq-restricted T-cell responses. Dose-response curves for binding to DR4. Dose-response curves for DR4-restricted T-cell responses. PLS Permutations experiments. RMSD vs. simulation time plots for the whole complex and the ligand. The distance between the GalHyl264 CA and the Aq Lys11C as a function of the simulation time. References. (PDF) [file pone.0017881.s001.pdf]

SUPPORTING INFORMATION

Design of Glycopeptides Used to Investigate Class II MHC Binding and T-Cell Responses Associated with Autoimmune Arthritis

Ida E. Andersson,[1#] C. David Andersson,[1#] Tsvetelina Batsalova,[2] Balik Dzhambazov,[2] Rikard Holmdahl,[2] Jan Kihlberg,[1,3] and Anna Linusson[3]*

[1] Department of Chemistry, Umeå University, SE-901 87 Umeå, Sweden. [2] Medical Inflammation Research, Department of Medical Biochemistry and Biophysics, Karolinska Institute, SE-171 77 Stockholm, Sweden. [3] AstraZeneca R&D Mölndal, SE-431 83 Mölndal, Sweden.

Contents

**S1-S2**	Contents
**S3-S4**	Amino acid descriptors
**S5**	Amino acid derivatives incorporated into virtual peptides at positions p260 and p263
**S6-S9**	Docking software parameter tuning and constraints
**S10**	Table of amino acids selected for p260 and p263 based on docking scores
**S11-12**	Score and loading plots from the PCA based on physicochemical properties of amino acids in p260 and p263
**S13**	Table with yields, purity and MALDI-TOF data for the synthesized glycopeptides
**S14-19**	HPLC chromatograms for the modified glycopeptides
**S20**	Initial A^q binding affinity assay
**S21**	Dose-response curves for binding to A^q
**S22**	Dose-response curves for A^q-restricted T-cell responses
**S23**	Dose-response curves for binding to DR4
**S24**	Dose-response curves for DR4-restricted T-cell responses

S25 PLS Permutations experiments

S26 RMSD vs. simulation time plots for the whole complex and the ligand

S27 The distance between the GalHyl^{264} CA and the A^{q} Lys11C as a function of the simulation time

S28 References

Table 1. Descriptors describing the amino acids in peptide position p260.

Descriptor*	Descriptor*	Descriptor*
Weight	logP(o/w)	chi0v
vdw_area	SlogP	chi0v_C
vdw_vol	logS	chi1v
vol	PEOE_PC+	chi1v_C
density	PEOE_RPC+	chi0
dens	PEOE_RPC-	chi0_C
glob	PEOE_VSA+0	chi1
diameter	PEOE_VSA-0	chi1_C
radius	PEOE_VSA_FHYD	VAdjEq
AM1_E	PEOE_VSA_FNEG	VAdjMa
AM1_Eele	PEOE_VSA_FPNEG	VDistEq
AM1_HF	PEOE_VSA_FPOL	VDistMa
AM1_HOMO	PEOE_VSA_FPOS	weinerPath
AM1_IP	PEOE_VSA_HYD	weinerPol
AM1_LUMO	PEOE_VSA_NEG	zagreb
a_count	PEOE_VSA_POS	balabanJ
a_IC	PC+	BCUT_PEOE_0
a_ICM	PC-	BCUT_PEOE_2
a_nH	RPC+	BCUT_PEOE_3
b_1rotN	RPC-	BCUT_SLOGP_0
b_1rotR	ASA	BCUT_SLOGP_3
b_count	ASA+	BCUT_SMR_0
b_rotN	ASA-	BCUT_SMR_1
b_rotR	ASA_H	BCUT_SMR_2
b_single	ASA_P	BCUT_SMR_3
a_heavy	CASA+	GCUT_PEOE_1
a_nC	CASA-	GCUT_PEOE_2
a_nF	DASA	GCUT_PEOE_3
a_nN	DCASA	GCUT_SLOGP_1
a_nO	FASA+	GCUT_SLOGP_2
a_nS	FASA-	GCUT_SLOGP_3
b_heavy	FASA_H	GCUT_SMR_0
a_acc	FASA_P	GCUT_SMR_1
a_don	FCASA+	GCUT_SMR_2
a_hyd	FCASA-	GCUT_SMR_3
Kier1	VSA	
Kier2	vsa_hyd	
Kier3	apol	
KierA1	bpol	
KierA2	dipole	
KierA3	pmi	
KierFlex	rgyr	
SMR	std_dim1	
mr	std_dim2	
	std_dim3	

Calculated in MOE 2008.10, Chemical Computing Group Inc., Suite 910 - 1010 Sherbrooke St. W, Montreal, Quebec, Canada H3A 2R7, **2008.*

Table 2. Descriptors describing the amino acids in peptide position p263.

Descriptor*	Descriptor*	Descriptor*
Weight	logP(o/w)	chi0v
vdw_area	SlogP	chi0v_C
vdw_vol	logS	chi1v
vol	PEOE_PC+	chi1v_C
density	PEOE_PC-	chi0
dens	PEOE_RPC+	chi0_C
glob	PEOE_RPC-	chi1
diameter	PEOE_VSA_FHYD	chi1_C
AM1_dipole	PEOE_VSA_FNEG	VAdjEq
AM1_E	PEOE_VSA_FPNEG	VAdjMa
AM1_Eele	PEOE_VSA_FPOL	VDistEq
AM1_HF	PEOE_VSA_FPOS	VDistMa
AM1_HOMO	PEOE_VSA_FPPOS	weinerPath
AM1_IP	PEOE_VSA_HYD	weinerPol
AM1_LUMO	PEOE_VSA_NEG	zagreb
a_count	PEOE_VSA_POS	balabanJ
a_IC	PC+	BCUT_PEOE_1
a_ICM	PC-	BCUT_PEOE_2
a_nH	RPC+	BCUT_SLOGP_1
b_1rotR	RPC-	BCUT_SLOGP_2
b_count	ASA	BCUT_SMR_1
b_rotR	ASA+	BCUT_SMR_2
b_single	ASA-	GCUT_PEOE_1
a_heavy	ASA_H	GCUT_PEOE_2
a_nBr	ASA_P	GCUT_PEOE_3
a_nC	CASA+	GCUT_SLOGP_0
a_nCl	CASA-	GCUT_SLOGP_1
a_nF	DASA	GCUT_SLOGP_2
a_nI	DCASA	GCUT_SLOGP_3
a_nN	FASA+	GCUT_SMR_1
a_nO	FASA-	GCUT_SMR_2
a_nS	FASA_H	GCUT_SMR_3
b_heavy	FASA_P	SMR
a_acc	FCASA+	mr
a_don	FCASA-	std_dim1
a_hyd	VSA	std_dim2
Kier1	vsa_hyd	std_dim3
Kier2	apol	KierFlex
Kier3	bpol	
KierA1	dipole	
KierA2	pmi	
KierA3	rgyr	

Calculated in MOE 2008.10, Chemical Computing Group Inc., Suite 910 - 1010 Sherbrooke St. W, Montreal, Quebec, Canada H3A 2R7, **2008.*

Figure 1. Amino acid derivatives incorporated into virtual peptides at positions p260 and p263 named by CAS number (Fmoc-protected amino acids) or supplier ID

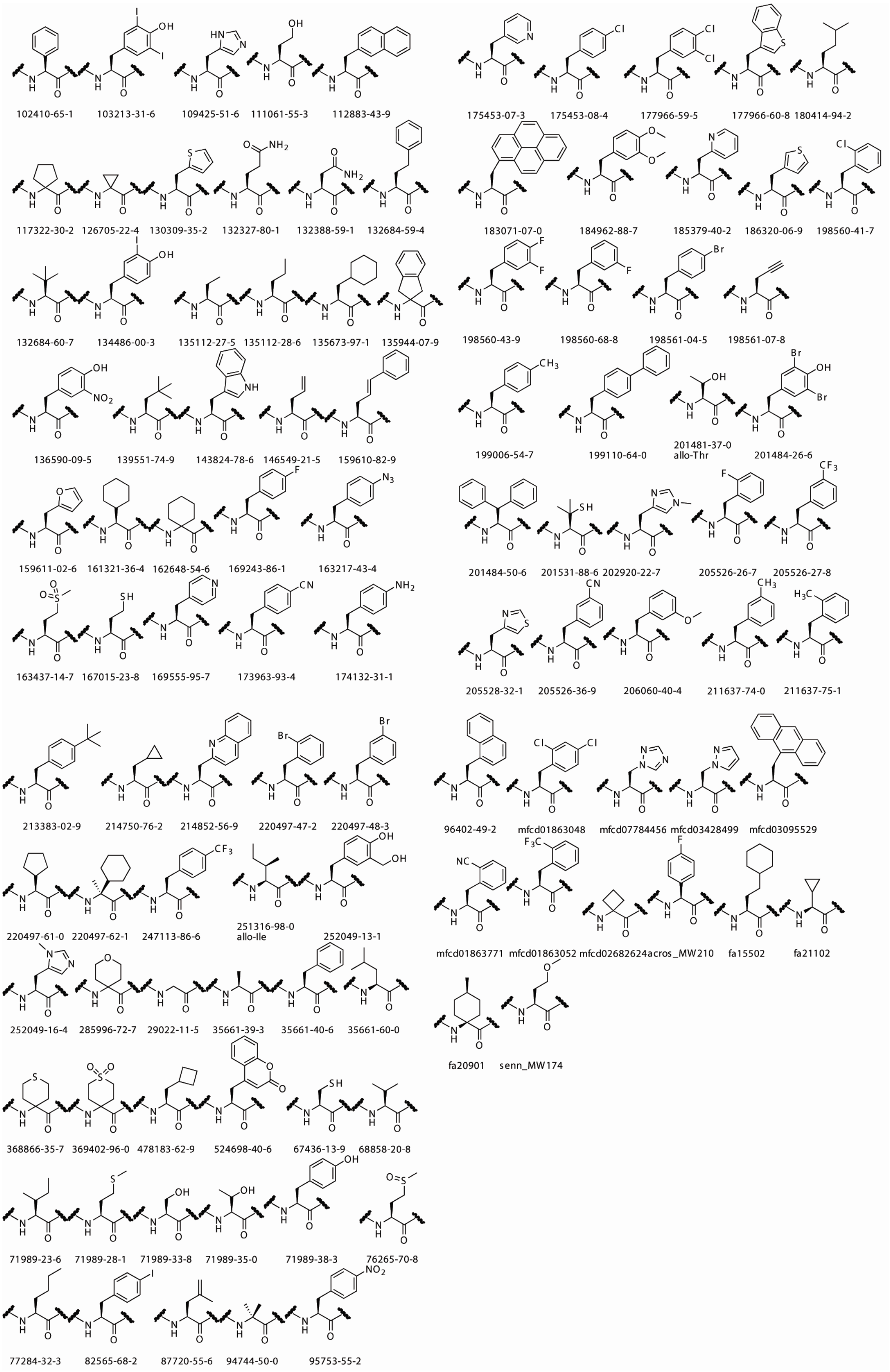

Docking software parameter tuning and constraint

INTRODUCTION

Initial docking experiments with A^q and the peptide sequence Ac-Ile^{260}-Ala-Gly-Phe^{263}-Lys-Gly-Glu-Gln^{267}-NH_2 (referred to as the original peptide) using default parameter settings in OMEGA [1] and FRED [2] revealed that the software were not able to reproduce the original peptide pose when docking a randomized low-energy conformation of the same. In order for a virtual screen (VS) to be fruitful we, and others [3], believe that the docking software used in the screening should be able to reproduce the binding mode and the X-ray ligand. Using OMEGA default parameters generated a conformations with an RMSD of 2.74 Å compared to the original peptide conformation. The altered settings resulted in conformations with an RMSD of 2.6 Å. In FRED the best suggested pose had an RMSD of 4.07 while the most resembling pose generated by OMEGA had an RMSD of 2.60 Å to the original peptide. The high RMSD values were mainly due to differences in the sequence Gly^{265}-Glu^{266}-Gln^{267}. RMSD between these residues and the corresponding residues in the original peptide was 5.71. The RMSD between the amino acids in position p260 to p263 (which anchor to MHC), was 1.66 Å. Furthermore, the results from dockings of a set of ten trial peptides revealed wrongly positioned anchoring residues. Visual studies of the docking solutions revealed that the suggested peptide pose did not interact with the protein at the two crucial anchoring points (P1 and P4 pockets in A^q), or that the peptide was turned in the binding pocket.

We have previously shown that altering docking software parameters can has significant effects on docking outcome and that statistical design is an excellent way of explore these effects [4]. Hence, software parameters were varied according to design of experiments (DoE) and the original peptide and the trial peptides were docked with constraints (see Methods in the main paper) using these different parameter settings.

METHODS

Docking constraints

A constraint was used to restrict the positioning of the Lys^{264} amino group in the docked peptide poses. Specifically, it was forced to always be projecting ‘out’ from the protein-binding cleft. A substructure of the Lys^{264} amino group represented by a Daylight SMART string [5] was given to FRED, which constrained the positioning of the amino group to a sphere around the original position.

Tuning of FRED docking parameters and OMEGA parameters

Peptide conformations docked using FRED were pre-generated using OMEGA[2] with settings tuned according to the findings of Kirchmair and co-workers [6] (Table 3). DoE was applied to investigate the effects on the docking outcome of adjustable parameters in FRED full factorial parameter design [7]. The aim was to relate the parameter settings to the root mean square deviation (RMSD) value between the original peptide from the comparative model of A^q and the resulting

conformations or docking poses. The full factorial design exhaustively generated combinations of all parameters and their respective settings resulting in N parameter sets according to $N = X^k$ where X was the number of parameter levels and k was the number of parameters. The chosen parameter values in FRED (Table 4) were varied in a three-level full factorial design using MODDE software [8].

A parameter screen was performed to ensure that FRED was able retrieve a conformation closely resembling the original peptide. The qualitative parameters *exhaustive score* and *optimization* (Table 4) had settings corresponding to scoring functions and the scoring functions included in this study were selected to be as different as possible in the way that they consider different types of molecular interactions. For example, Shapegauss[9] does not consider hydrogen bonds contrary to Chemgauss3, Plp [10], CGO and Screenscore [11]. Chemgauss3 considers desolvation energies but the other scoring functions do not. The parameters presented in Table 4 were subjected to a full factorial design resulting in 64 (4^3) parameter settings. The conformational library of the original peptide generated by OMEGA (see the Introduction) was docked in FRED using all 64 settings generated by the factorial design and the default setting. The final scoring function was Chemgauss3 (default).

Table 3. Tuned OMEGA-settings.

OMEGA parameter	Tuned setting
ewindow	0.25
rms	0.6
maxconf	1000
buildff	Mmff94s_Trunc

Table 4. FRED Parameters Subjected to Design of Experiments.

level	exhaustive score	optimization	clash scale[b]
1	chemgauss3[a]	chemgauss3[a]	0.25
2	shapegauss	shapegauss	0.5
3	plp	plp	0.7[c]
4	cgo[c]	screenscore	1.0

[a]Default setting. [b]clash scale has no default setting. [c]Tuned settings.

Evaluation of Docking Parameter Tuning

The top pose for each docking was evaluated by calculation of RMSD to the original peptide. The parameter settings resulting in the best dockings (*i.e.* low RMSD to original peptide) were further evaluated by an additional docking run using the original peptide and control runs using a set of ten trial peptides (Table 5). These dockings were evaluated by visual inspection of the positioning of the amino acid side chains in p260 and p263 and by RMSD calculations between the peptide backbone of the 260-263 fragment to the original peptide structure. A docking was considered successful when both these side chains pointed down into their respective protein cavity pockets with a backbone RMSD < 3 Å.

RESULTS AND DISCUSSION

Parameters were tuned in OMEGA inspired by results presented by Kirchmair *et al.* [6] which indicated that a high setting on *ewindow* (*i.e.* 25 kcal/mol or above in OMEGA version 2.0) was desirable, or else, valuable conformations were at risk of being rejected in the final conformation ensemble. The same study indicated that a low setting on the *rms* parameter was beneficial for low RMSD to crystal conformations. Hence, OMEGA was run with settings according to Table 3 and all other parameters were kept at their default values. Subsequent experiments with OMEGA, not included in the present study, where parameters were varied according to a statistical experimental design resulted in conformations with an RMSD of 1.6 to the original peptide.

Tuning of parameters in FRED Table 4. For FRED, different settings on the parameter *exhaustive score* influenced the results the most. Setting *clash scale* to 1.0 gave no docking solutions. The parameter *optimization* had little or no effect on the results and we decided to turn off optimization to save time in the extensive FRED VS. The tuned settings in FRED resulted in docking solutions equal to default setting docking solutions for the original peptide but improved results for the ten trial peptides (Table 5), in terms of backbone RMSD of the p260-p263 fragment. The reason for *exhaustive scoring* CGO, used in the tuned settings, being able to rank good poses high is possible due to the nature of the scoring function where the shape of the ligand is matched to the original ligand. RMSD values of the peptide backbone top scored trial peptides versus the backbone of the original peptide are presented in Table 5. Visual inspections of docking poses revealed that, in general, an RMSD higher than 8 Å indicated incorrectly turned peptides and an RMSD less than 3 Å indicated peptides where amino acids in p260 and p263 were in the vicinity of their anchor-positions. Applying the tuned docking settings, all top ranked docking poses were correctly turned in the A^q binding site (Table 5) as opposed to the default settings where three out of ten were incorrectly turned. Finally, an additional benefit of using the tuned settings was a slight reduction in time consumption for each docking.

Table 5. Table of trial peptide backbone p260-p263 fragment RMSD (compared to the original ligand) docked with default and tuned docking parameters in FRED.

Peptide	p260	p263	FRED default RMSD (Å)	FRED tuned RMSD (Å)
original	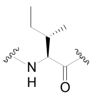	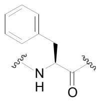	1.6	1.8
1	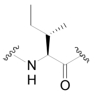	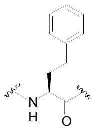	2.8	2.8
2	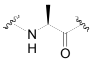	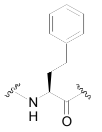	15.9	4.48
3	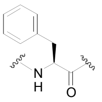	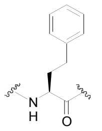	3.2	3.58
4	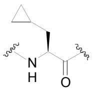	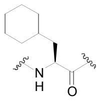	0.9	1.0
5	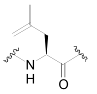	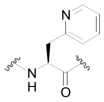	5.7	6.0
6	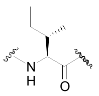	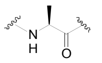	1.7	1.7
7	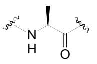	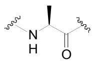	14.9	5.9
8	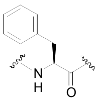	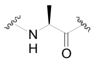	14.0	5.4
9	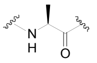	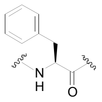	6.2	6.5
10	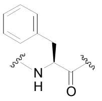	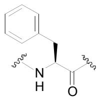	5.8	5.6

Table 6. Amino acids selected for p260 based on docking scores. Corresponding molecular structures can be found in Figure 1.

CAS name	CAS name	CAS name	CAS name	Supplier ID
102410-65-1	159611-02-6	214750-76-2	71989-28-1	acros_MW210
109425-51-6	161321-36-4	220497-61-0	76265-70-8	fa21102
111061-55-3	163437-14-7	220497-62-1	77284-32-3	mfcd03428499
117322-30-2	167015-23-8	251316-98-0	87720-55-6	senn_MW174
130309-35-2	175453-07-3	252049-16-4	109425-51-6_taut#	
132327-80-1	180414-94-2	35661-60-0		
132684-59-4	186320-06-9	478183-62-9		
132684-60-7	198561-07-8	67436-13-9		
135112-28-6	201531-88-6	68858-20-8		
135944-07-9	202920-22-7	71989-23-6		
139551-74-9	205528-32-1	35661-60-0		

One additional tautomeric form of histidine

Table 7. Amino acids selected for p263 based on docking scores. Corresponding molecular structures can be found in Figure 1.

CAS name	CAS name	CAS name	CAS name	CAS name	Supplier ID
103213-31-6	169555-95-7	198560-41-7	205526-36-9	252049-13-1	mfcd01863048
109425-51-6	173963-93-4	198560-43-9	205528-32-1	252049-16-4	mfcd01863771
112883-43-9	174132-31-1	198560-68-8	206060-40-4	35661-40-6	mfcd03428499
130309-35-2	175453-07-3	198561-04-5	211637-74-0	71989-38-3	
134486-00-3	175453-08-4	199006-54-7	211637-75-1	95753-55-2	
135673-97-1	177966-59-5	201484-26-6	213383-02-9	96402-49-2	
136590-09-5	177966-60-8	201484-50-6	214852-56-9	109425-51-6_taut#	
143824-78-6	184962-88-7	202920-22-7	220497-47-2		
159611-02-6	185379-40-2	205526-26-7	220497-48-3		
169243-86-1	186320-06-9	205526-27-8	247113-86-6		

One additional tautomeric form of histidine

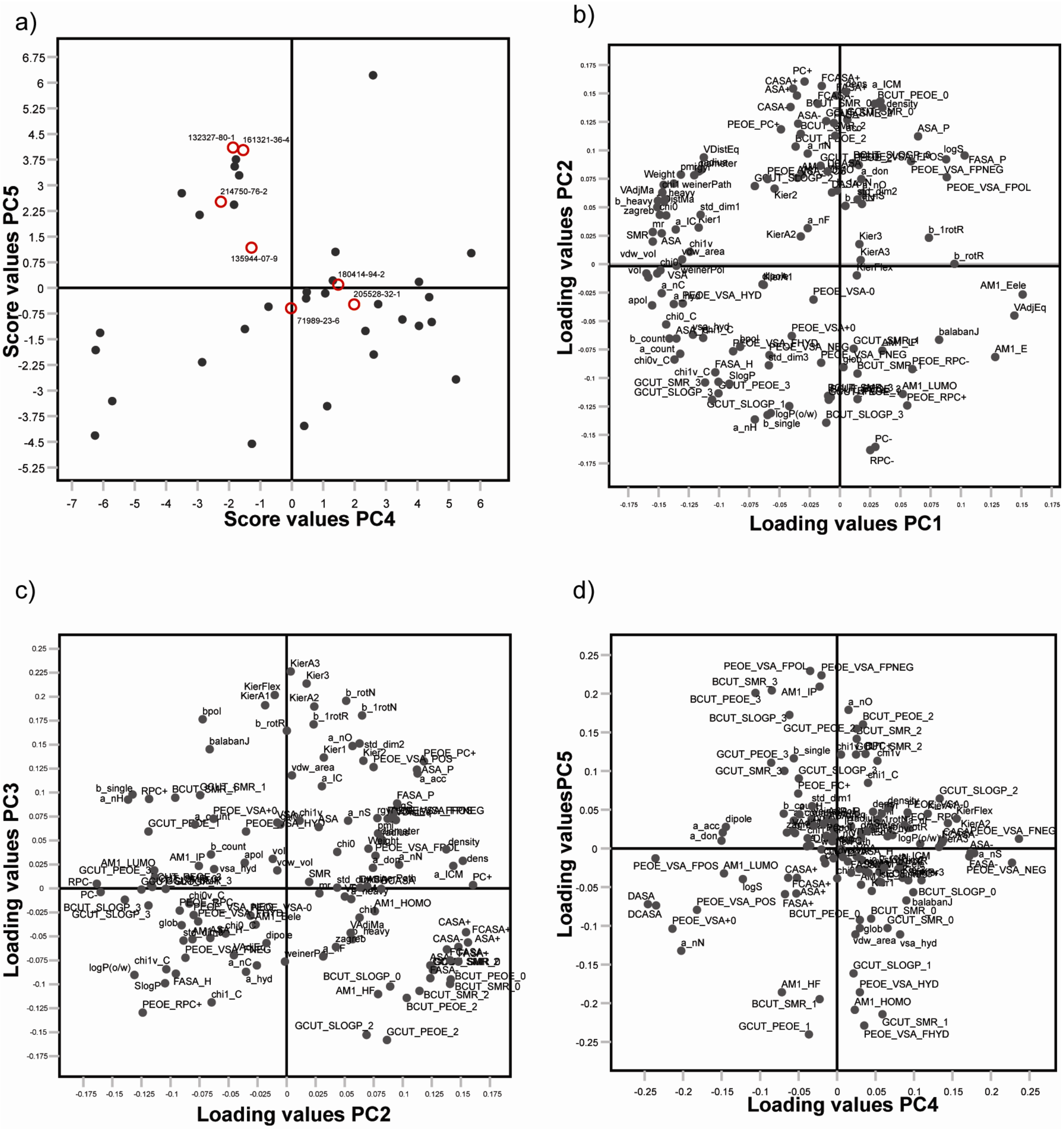

Figure 3. Score and loading plots for p260 amino acids from the PCA based on physicochemical properties. a) Score values of PC4 versus PC5. Selected amino acids are indicated by red circles. b) Loading values of PC1 versus PC2. c) Loading values of PC2 versus PC3. d) Loading values of PC4 versus PC5.

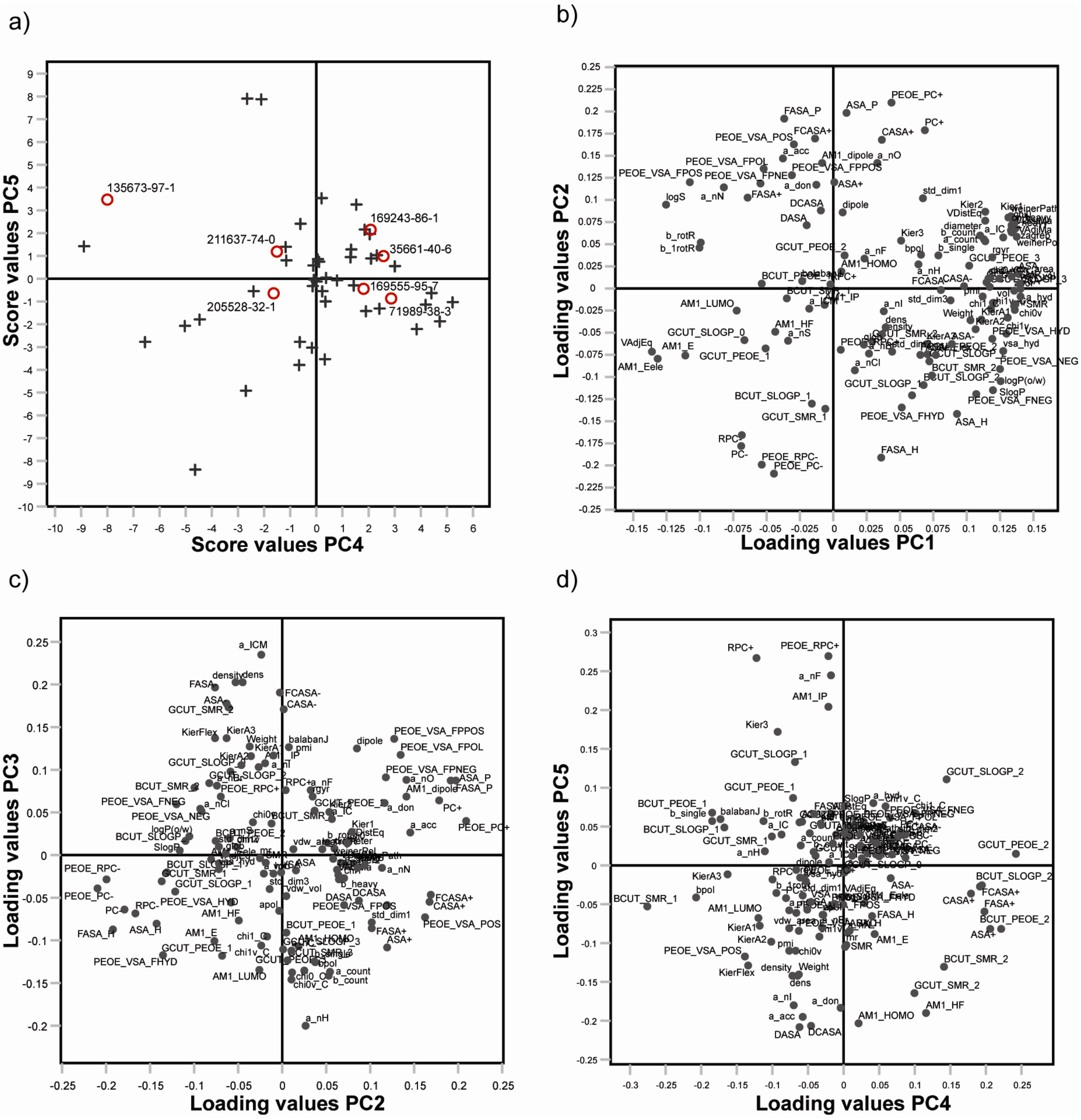

Figure 4. Score and loading plots for p263 amino acids from the PCA based on physicochemical properties. a) Score values of PC4 versus PC5. Selected amino acids are indicated by red circles. b) Loading values of PC1 versus PC2. c) Loading values of PC2 versus PC3. d) Loading values of PC4 versus PC5.

Table 8. Glycopeptide characterization.

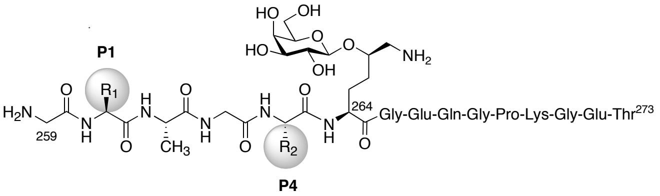

Glycopeptide	R_1	R_2	Isolated yield	HPLC purity (%)	MALDI-TOF Calculated mass $[M+H]^+/[M+Na]^+$	MALDI-TOF Observed mass $[M+H]^+/[M+Na]^+$
**2**	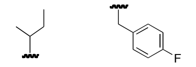		32.6 mg (40 %)	>99	1671.79/1693.77	1671.83/1693.75
**3**	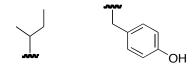		34.5 mg (42%)	>99	1669.79/1691.77	1669.79/1691.73
**4**	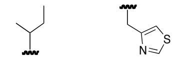		38.1 mg (47 %)	>99	1660.75/1682.73	1660.79/1682.72
**5**	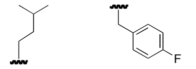		35.7 mg (43 %)	>99	1685.80/1707.78	1685.84/1707.77
**6**	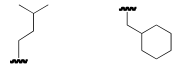		24.2 mg (29 %)	>99	1673.86/1695.84	1673.86/1695.80
**7**	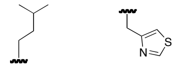		33.5 mg (41%)	>99	1674.76/1696.75	1674.78/1696.70
**8**	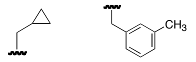		31.3 mg (38 %)	>99	1665.80/1687.78	1665.76/1687.71
**9**	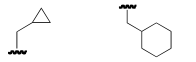		31.4 mg (39 %)	>97	1657.83/1679.81	1657.80/1679.77
**10**	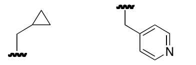		31.5 mg (39 %)	>97	1652.78/1674.76	1652.85/1674.73
**11**	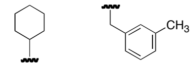		40.2 mg (48 %)	>99	1693.83/1715.81	1693.87/1715.80
**12**	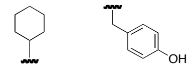		36.1 mg (46 %)	>99	1695.81/1717.79	1695.83/1717.74
**13**	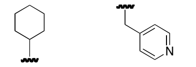		36.8 mg (44 %)	>99	1680.81/1702.79	1680.86/1702.79
**14**	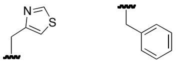		38.5 mg (46 %)	>99	1694.73/1716.71	1694.73/1716.65
**15**	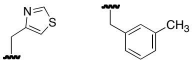		36.7 mg (44 %)	>99	1708.75/1730.73	1708.77/1730.70
**16**	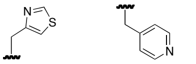		42.1 mg (51 %)	>99	1695.73/1717.71	1695.77/1717.68
**17**	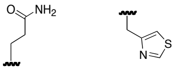		39.2 mg (47 %)	>99	1675.72/1697.70	1675.75/1697.67
**18**	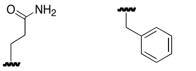		25.7 mg (30 %)	>99	1668.77/1690.75	1668.77/1690.71
**19**	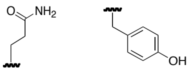		28.0 mg (34 %)	>99	1684.77/1706.75	1684.76/1706.69
**20**	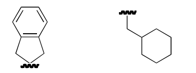		22.0 mg (26 %)	>99	1705.73/1727.81	1705.89/1727.83
**21**	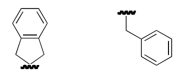		22.8 mg (27 %)	>99	1699.78/1721.76	1699.84/1721.78

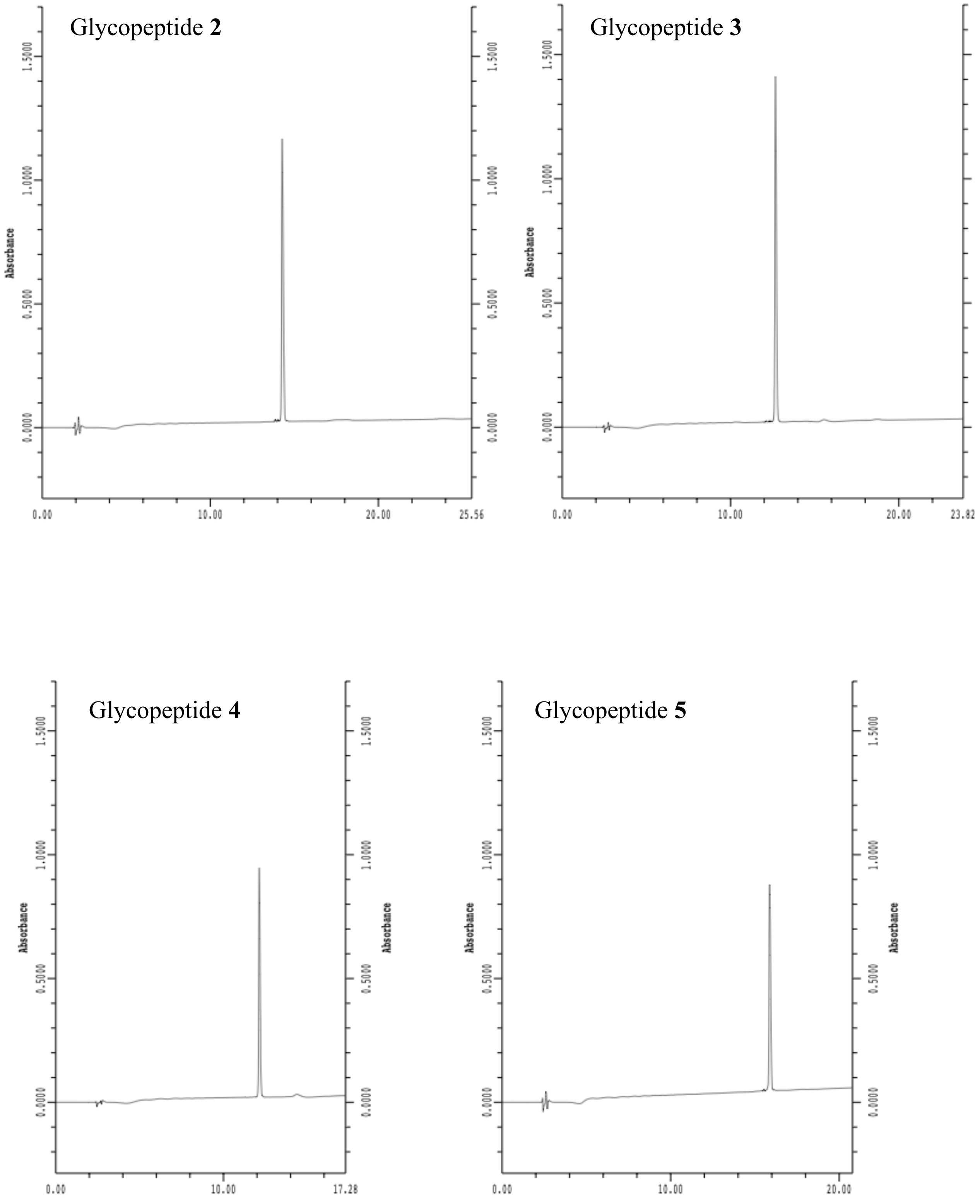

Figure 5. HPLC chromatograms for glycopeptides **2-5**.

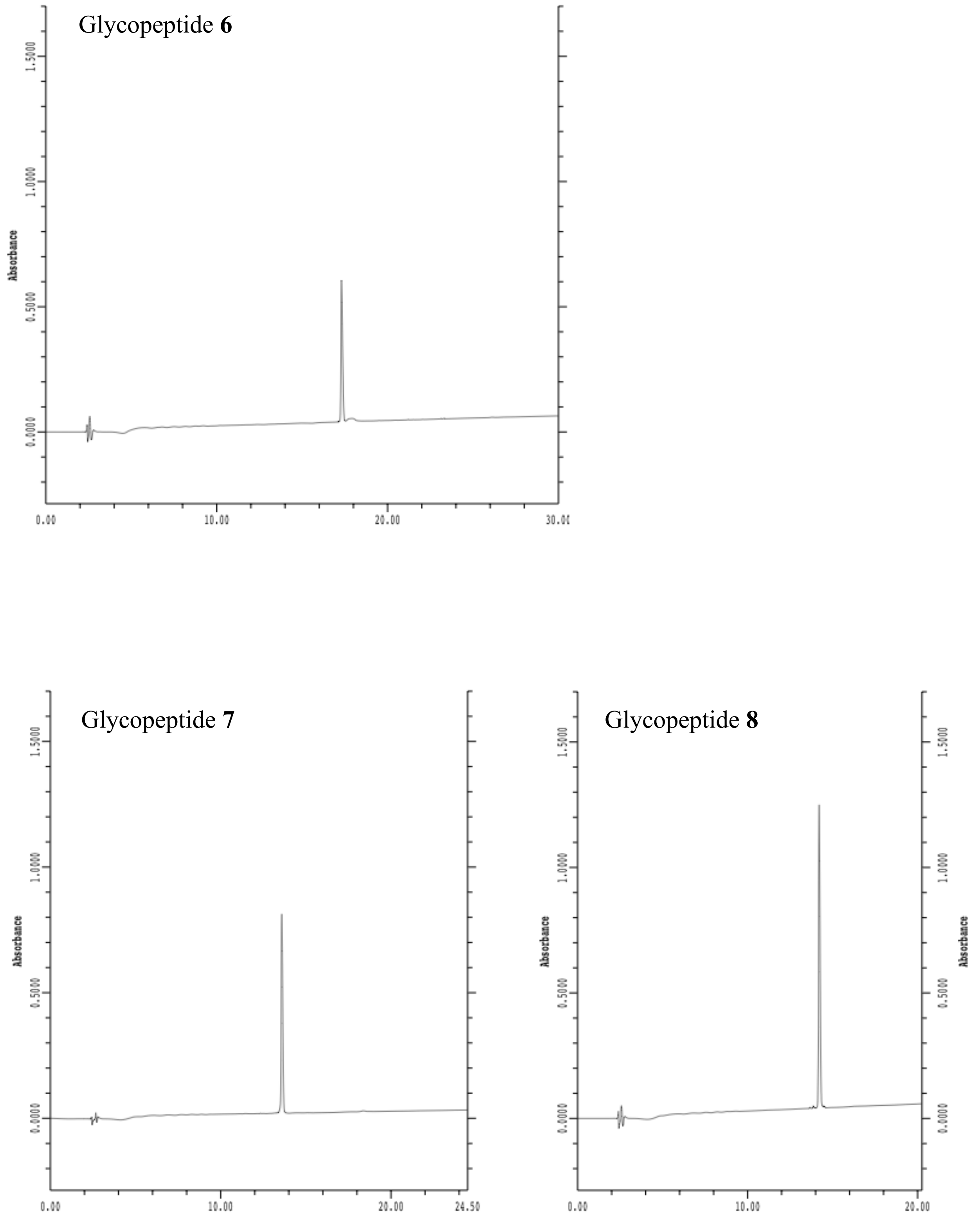

Figure 6. HPLC chromatograms for glycopeptides **6-8**.

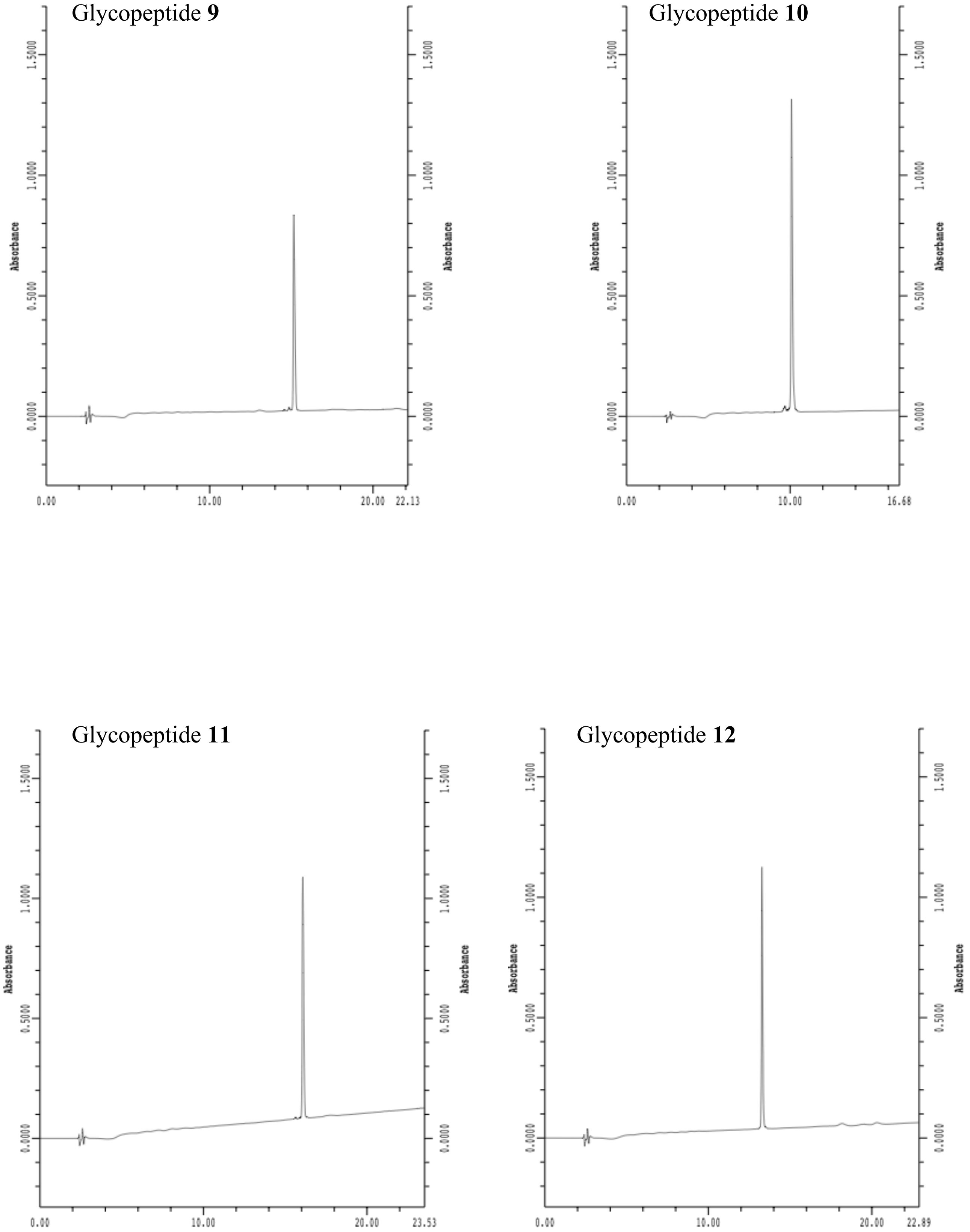

Figure 7. HPLC chromatograms for glycopeptides **9**-**12**.

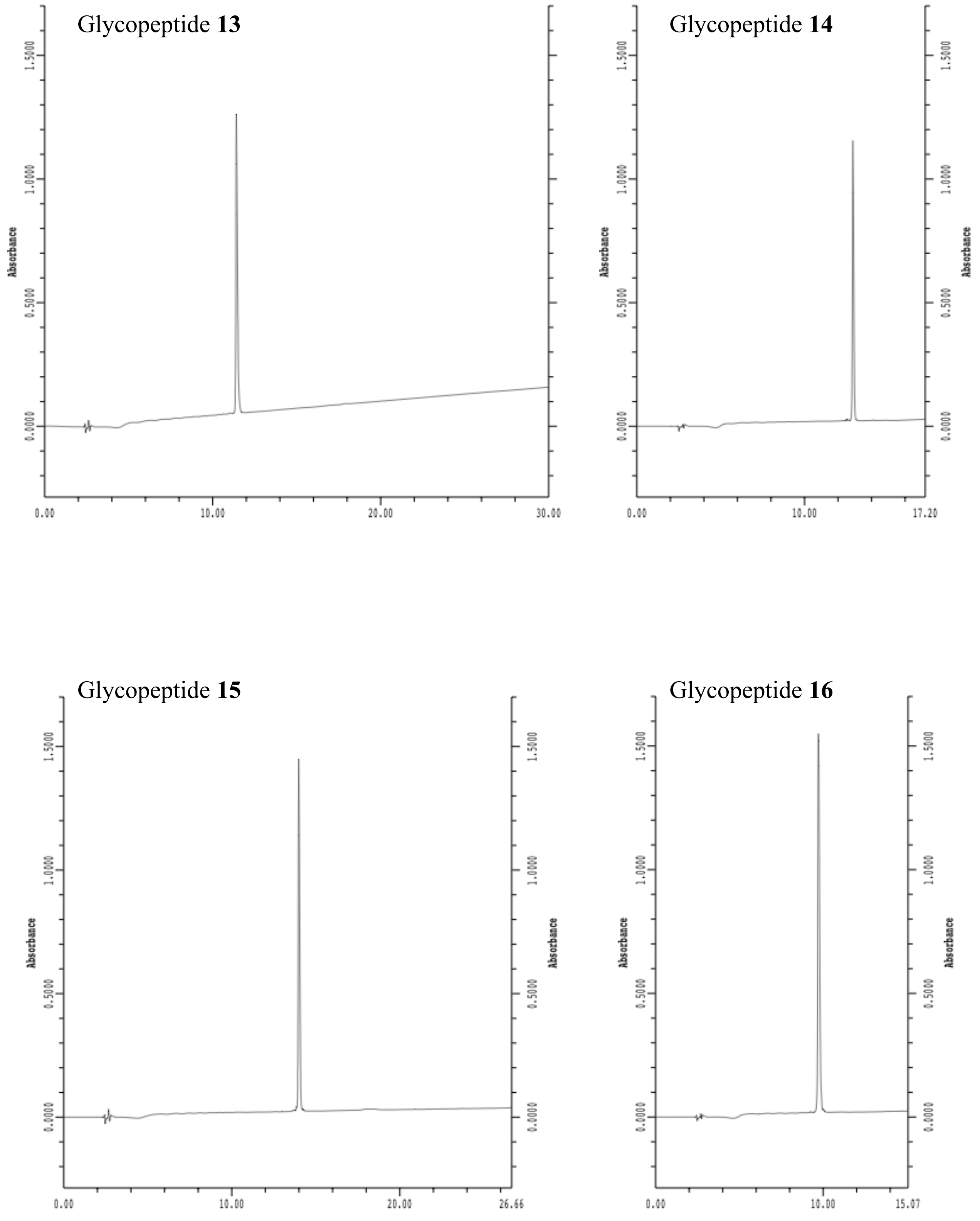

Figure 8. HPLC chromatograms for glycopeptides **13**-**16**.

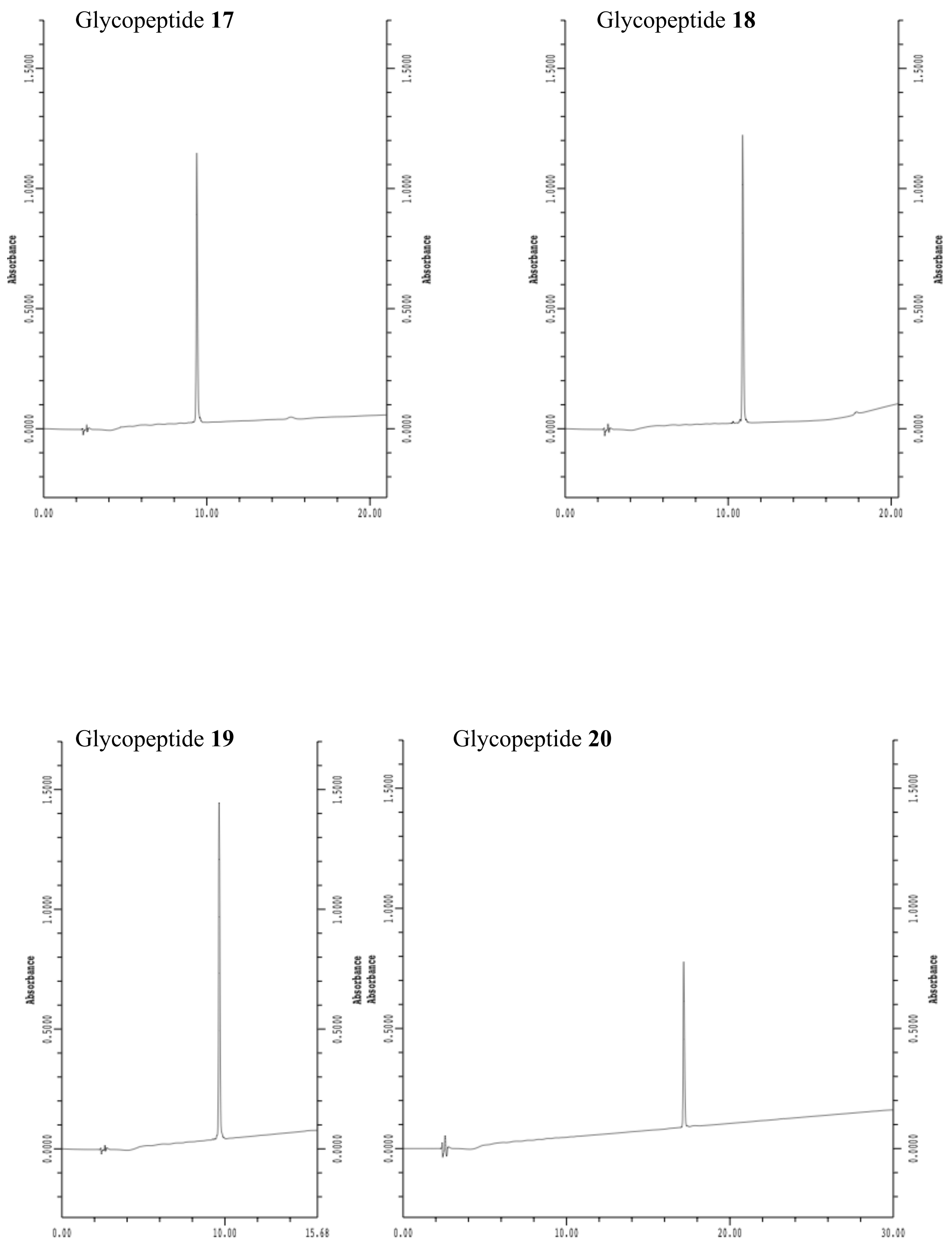

Figure 9. HPLC chromatograms for glycopeptides **17**-**20**.

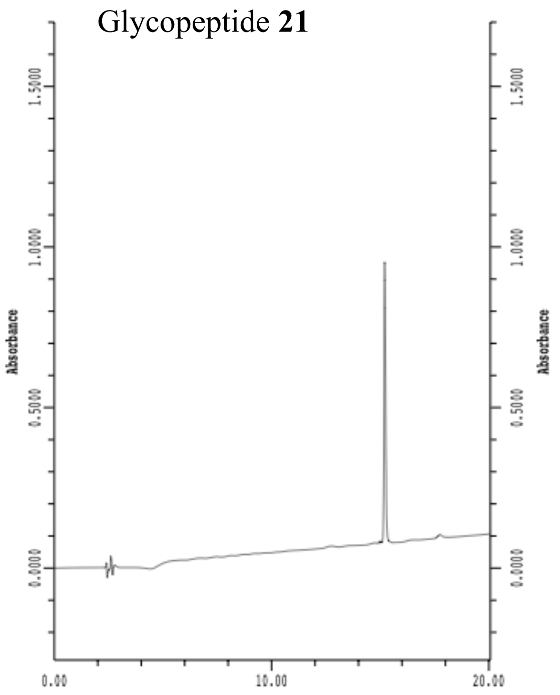

Figure 10. HPLC chromatograms for glycopeptides **21**.

Table 9. A^q binding assay performed in order to identify active and inactive glycopeptides by competitive inhibition of biotinylated CII259-273 binding to the A^q protein by **1**-**21**. The assay was performed with six antigen concentrations (0.16-500 μM) but % inhibition is only reported for 500 μM, which was the concentration used to define active glycopeptide (*i.e.* glycopeptides that showed more than 30% inhibition at 500 μM).[a]

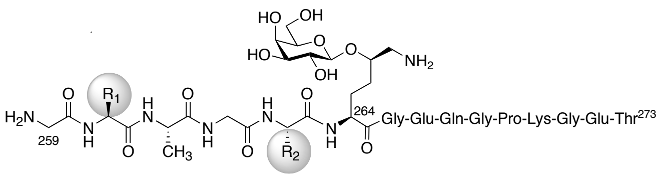

Peptide	R_1	R_2	% inhibition (500 μM)	Peptide	R_1	R_2	% inhibition (500 μM)
**1**	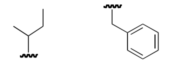		85 ± 3	**12**	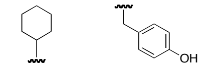		1 ± 4
**2**	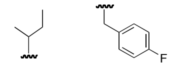		96 ± 1	**13**	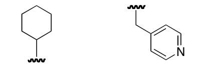		1 ± 5
**3**	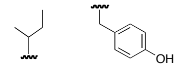		54 ± 5	**14**	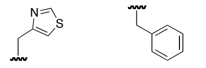		53 ± 3
**4**	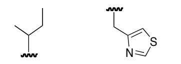		91 ± 0	**15**	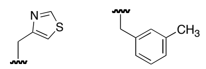		67 ± 1
**5**	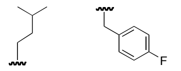		48 ± 4	**16**	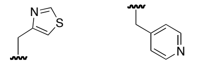		-37 ± 9
**6**	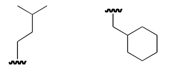		69 ± 6	**17**	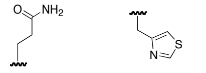		2 ± 10
**7**	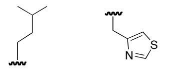		55 ± 10	**18**	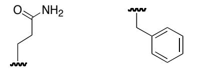		11 ± 5
**8**	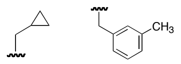		80 ± 5	**19**	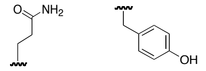		22 ± 3
**9**	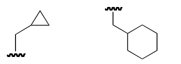		67 ± 1	**20**	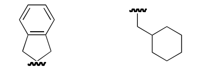		-9 ± 11
**10**	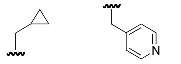		15 ± 7	**21**	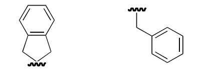		1± 6
**11**	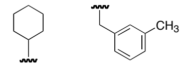		44 ± 1				

[a] Data are expressed as the percentage of biotinylated CII259-273 bound in the absence of competitor glycopeptide and are represented as mean values of triplicates ± one standard deviation.

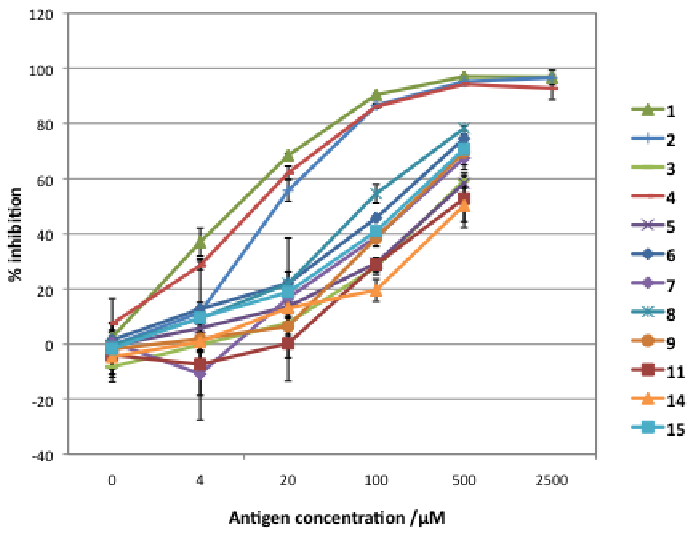

Figure 11. Inhibition of binding of a fixed concentration of biotinylated CII259-273 Lys^{264} peptide to recombinant A^{q} protein upon incubation with increasing concentrations of non-modified CII259-273 (**1**) or the modified glycopeptides **2-9**, **11**, and **14-15**, respectively. A^{q} bound biotinylated CII259-273 Lys^{264} peptide was detected in a time-resolved fluoroimmunoassay using europium labeled streptavidin. The points represent the average of triplicates and error bars are set to ± one standard deviation.

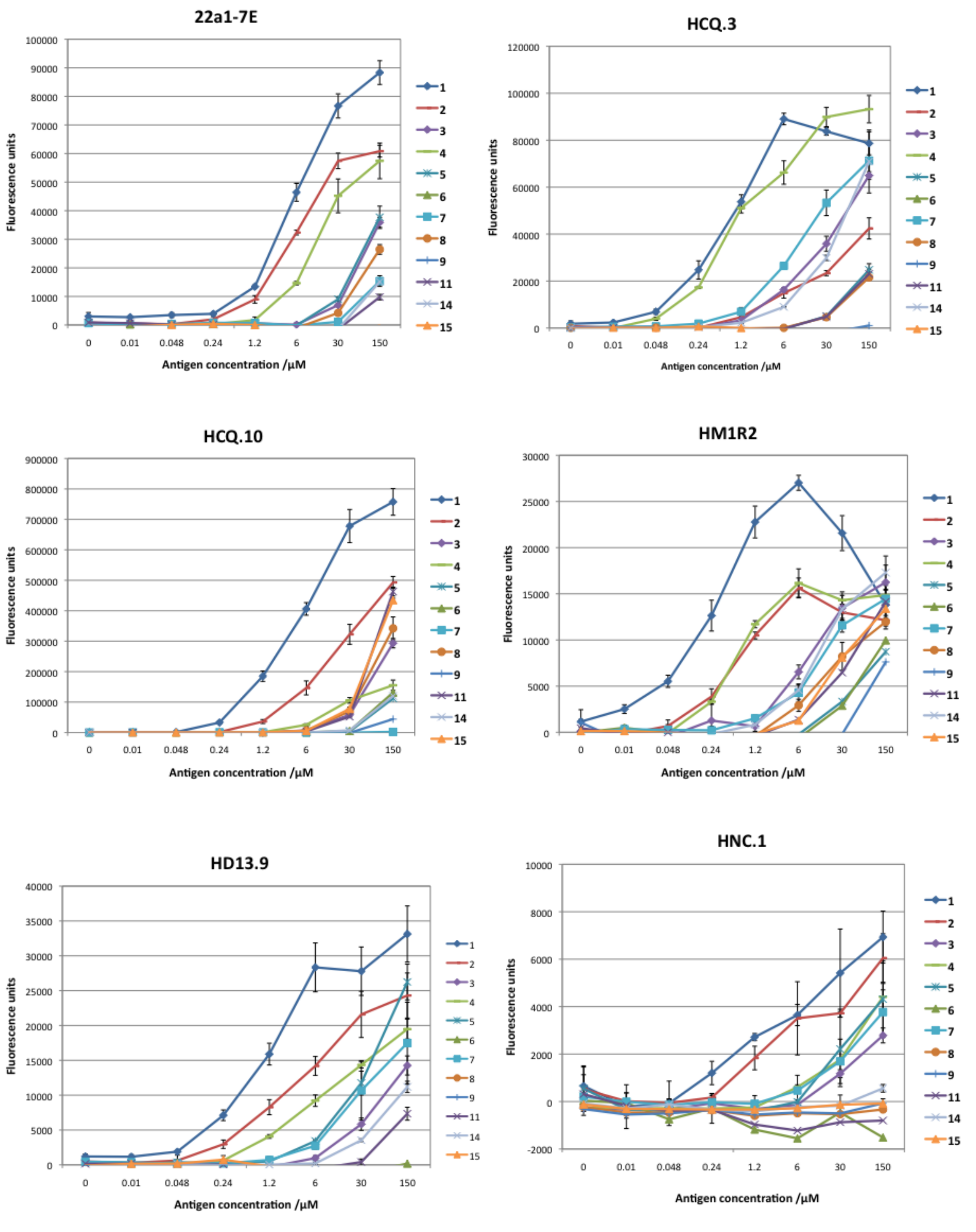

Figure 12. Response of A^{q}-restricted T-cell hybridomas after incubation with syngeneic spleen cells and increasing concentrations of non-modified CII259-273 (**1**) or the modified glycopeptides **2-9**, **11**, and **14-15**, respectively. Glycopeptide binding to A^{q} proteins on the spleen cells allows recognition by the T-cell hybridoma resulting in IL-2 secretion into the supernatant. Secreted IL-2 was subsequently quantified by a sandwich ELISA using the DELFIA system. The points represent the average of triplicates and error bars are set to ± one standard deviation.

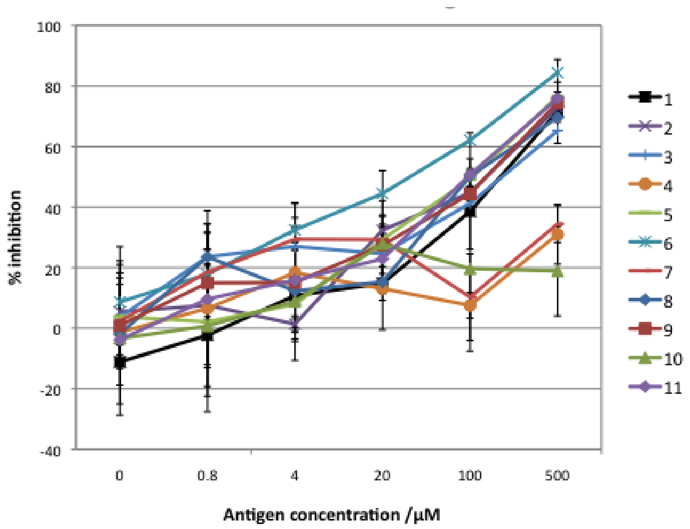

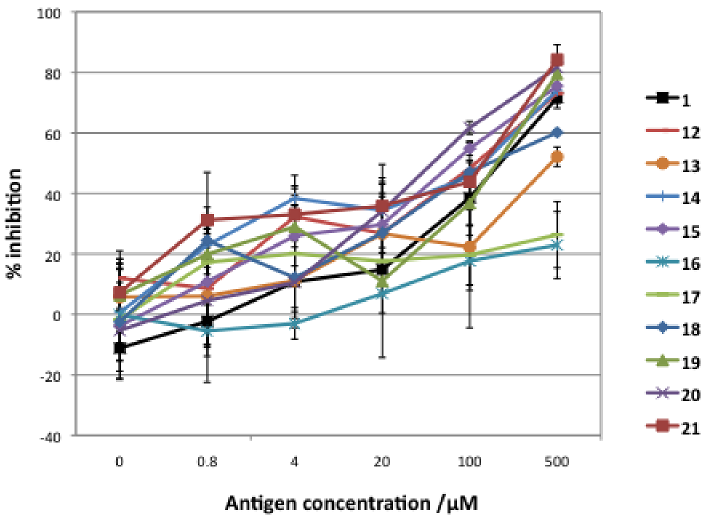

Figure 13. Inhibition of binding of a fixed concentration of biotinylated CLIP peptide to recombinant DR4 protein upon incubation with increasing concentrations of non-modified CII259-273 (**1**) or the modified glycopeptides **1-21**. DR4 bound biotinylated CII259-273 Lys^{264} peptide was detected in a time-resolved fluoroimmunoassay using europium labeled streptavidin. The points represent the average of triplicates and error bars are set to ± one standard deviation.

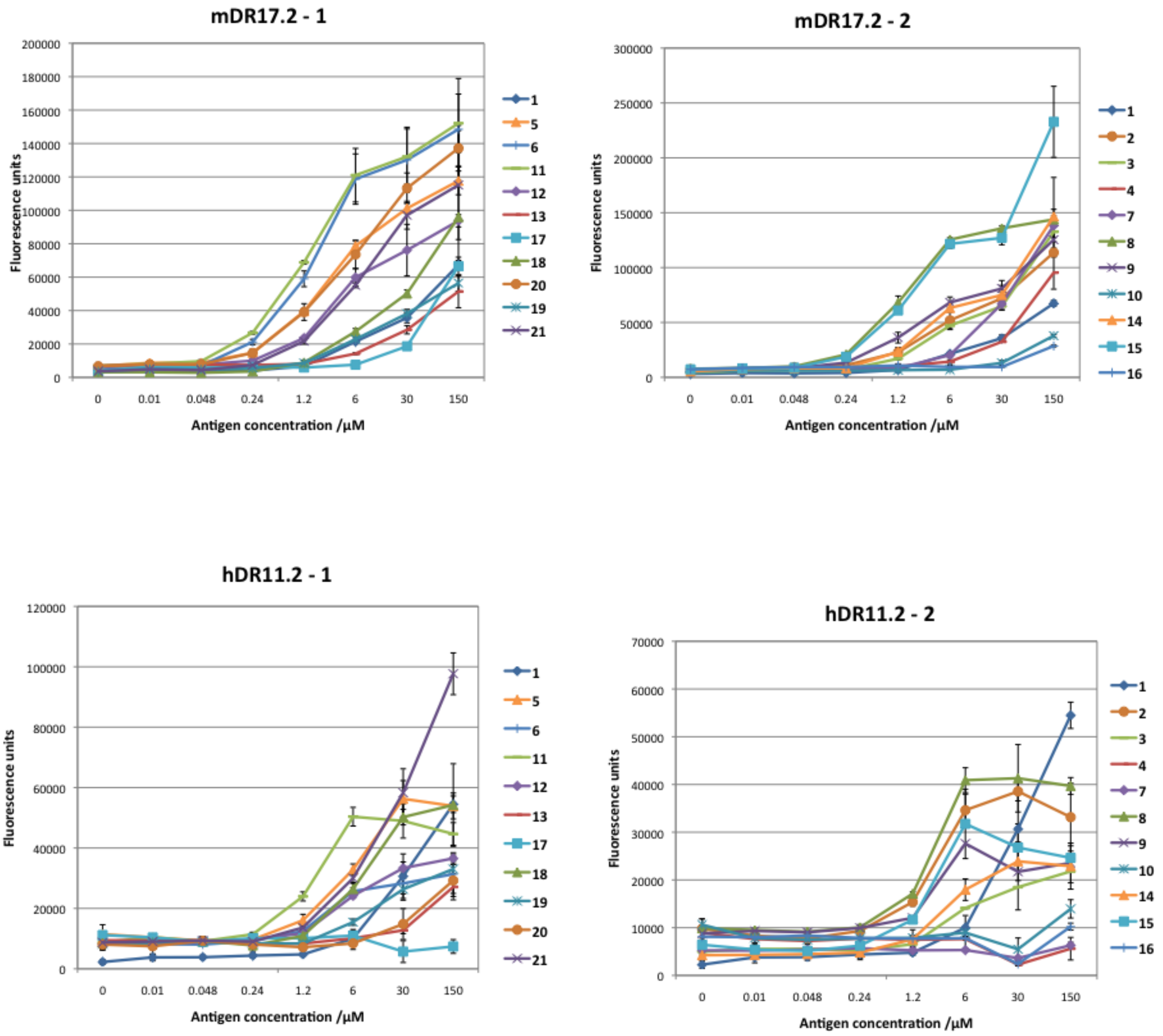

Figure 14. Response of DR4-restricted T-cell hybridomas after incubation with syngeneic spleen cells and increasing concentrations of glycopeptides **1**-**21**, respectively. Glycopeptide binding to DR4 proteins on the spleen cells allows recognition by the T-cell hybridoma resulting in IL-2 secretion into the supernatant. Secreted IL-2 was subsequently quantified by a sandwich ELISA using the DELFIA system. The points represent the average of triplicates and error bars are set to ± one standard deviation.

a) b)

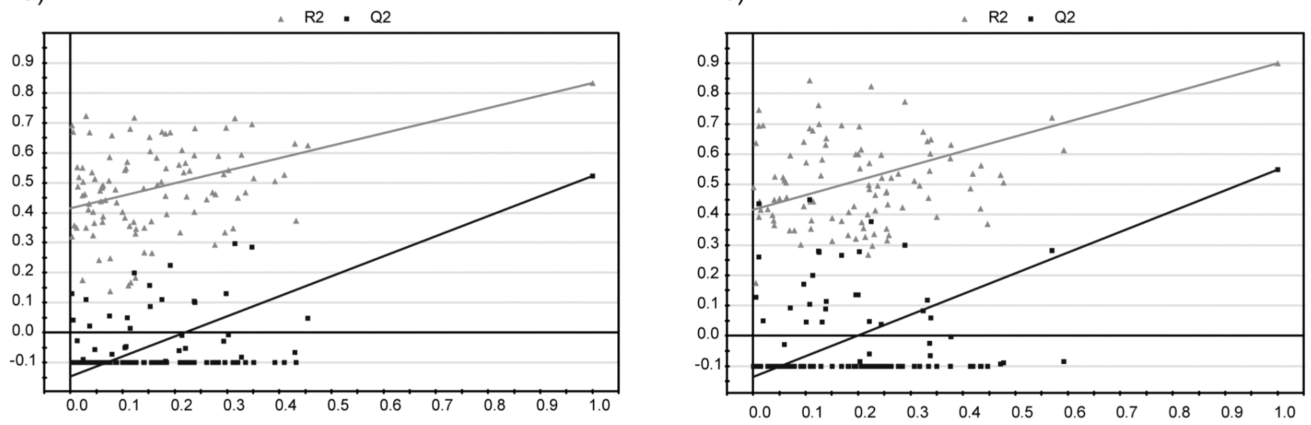

Figure 15. Permutation tests where the order of response-values have been permutated 100 times and R2 and Q2 for these 100 PLS-models have been plotted against the similarity between the original Y and the permutated Y. a) Results from the Aq PLS model. b) Results from the DR4 PLS models.

a) **Complex (protein + ligand) rmsd**

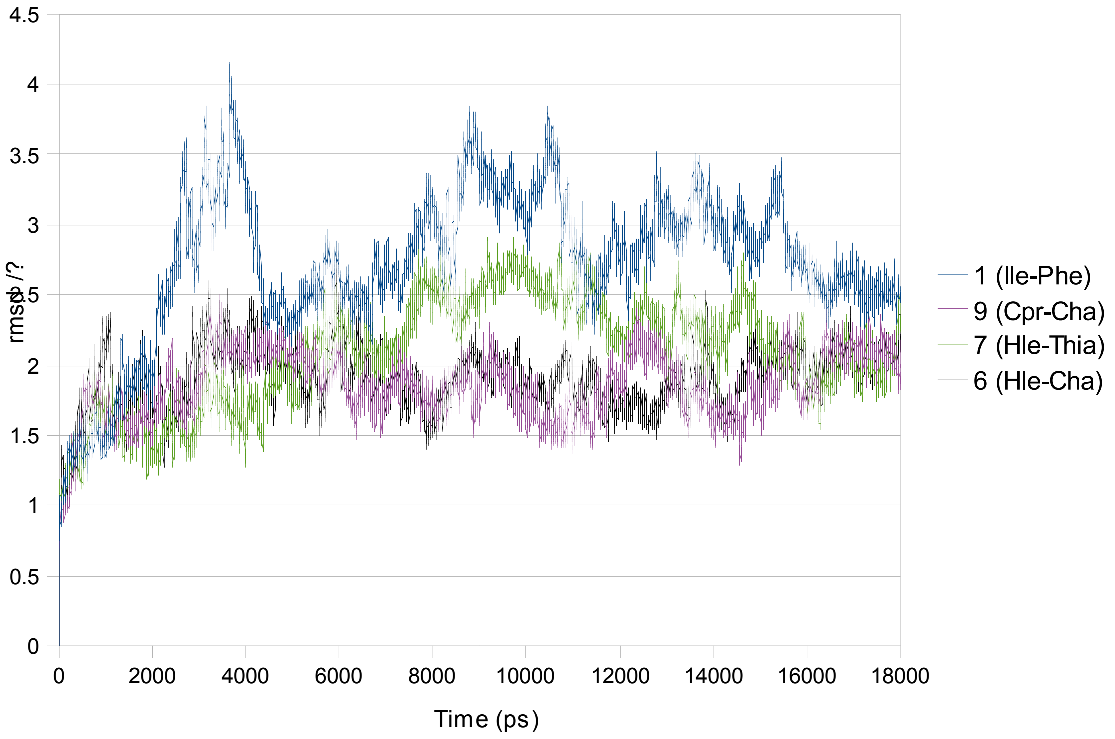

b) **Ligand backbone rmsd**

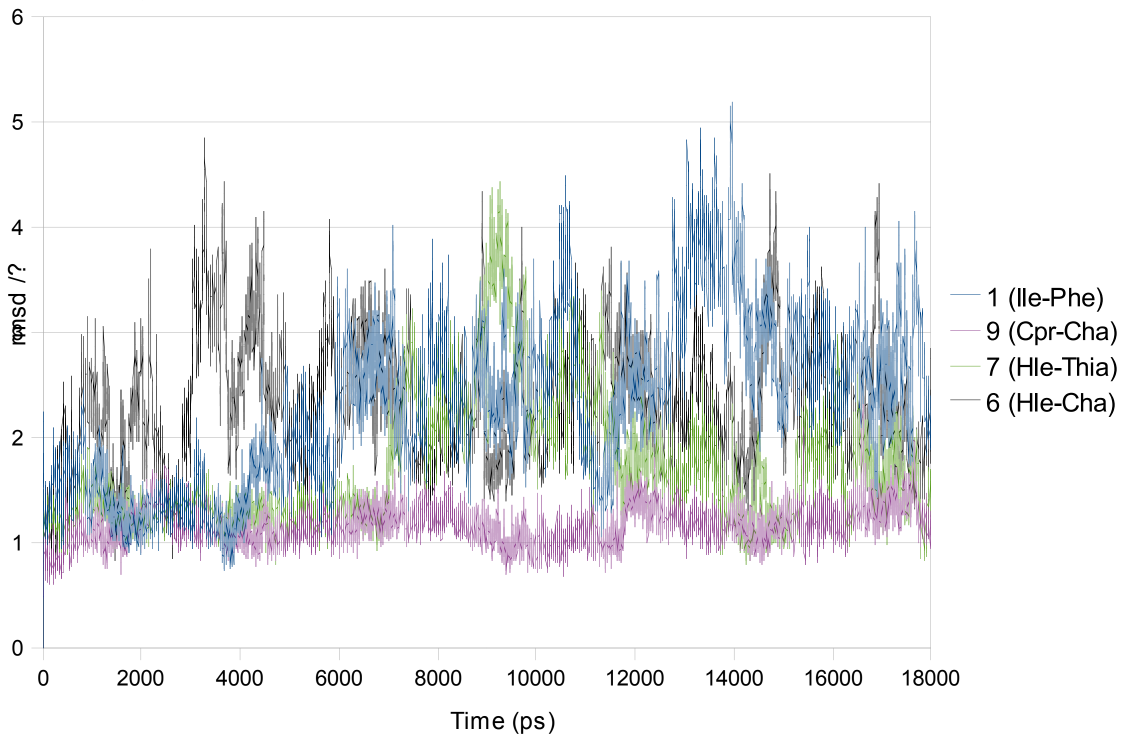

Figure 16. RMSD plotted as a function of the simulation time for a) the complex (*i.e.* the α_1 and β_1 domains of A^q and the glycopeptide) backbone C and N atoms or b) the glycopeptide backbone C and N atoms.

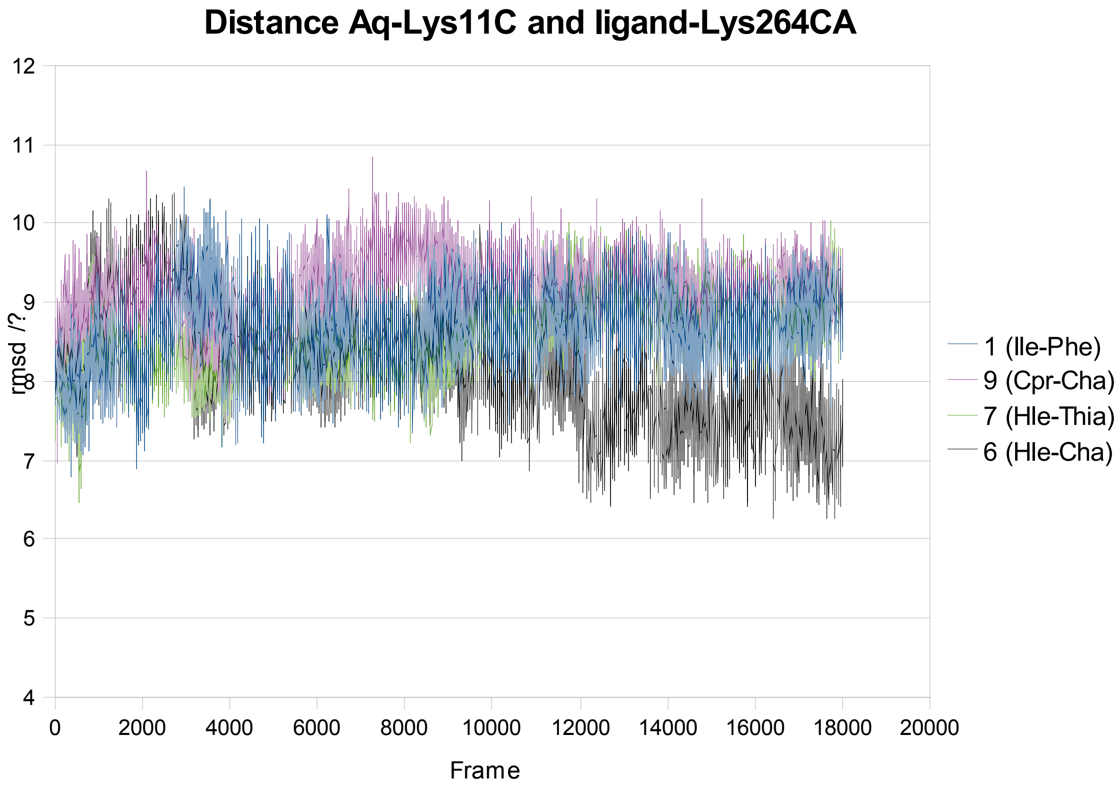

Figure 17. The distance between the GalHyl^{264} CA and the A^{q} Lys11C as a function of the simulation time.

References

1. OMEGA (2007) 2.2.0. OpenEye Scientific Software Inc. 3600 Cerrillos Road, Suite 1107, Santa Fe, NM 87507.
2. FRED (2007) 2.2.3. Openeye Scientific Software Inc. 3600 Cerrillos Road, Suite 1107, Santa Fe, NM 87507.
3. Verdonk ML, Berdini V, Hartshorn MJ, Mooij WTM, Murray CW, et al. (2004) Virtual screening using protein-ligand docking: Avoiding artificial enrichment. J Chem Inf Comput Sci 44: 793-806.
4. Andersson CD, Thysell E, Lindström A, Bylesjö M, Raubacher F, et al. (2007) A multivariate approach to investigate docking parameters' effects on docking performance. J Chem Inf Model 47: 1673-1687.
5. Jones G, Willett P, Glen RC, Leach AR, Taylor R (1997) Development and validation of a genetic algorithm for flexible docking. J Mol Biol 267: 727-748.
6. Kirchmair J, Wolber G, Laggner C, Langer T (2006) Comparative performance assessment of the conformational model generators omega and catalyst: A large-scale survey on the retrieval of protein-bound ligand conformations. J Chem Inf Model 46: 1848-1861.
7. Box GE, Draper NR (1987) Empirical Model-Building and Respons Surfaces. New York: John Wiley & sons, Inc.
8. MODDE (2008) 8.0.2. Umetrics AB. Box 7960, Umeå, Sweden.
9. McGann MR, Almond HR, Nicholls A, Grant JA, Brown FK (2003) Gaussian docking functions. Biopolymers 68: 76-90.
10. Verkivker GM, Bouzida D, Gehlaar DK, Rejto PA, Arthurs S, et al. (2000) Deciphering common failures in molecular docking of ligand-protein complexes. J Comput-Aided Mol Des 14: 731-751.
11. Stahl M, Rarey M (2001) Detailed analysis of scoring functions for virtual screening. J Med Chem 44: 1035-1042.
